# Genome Instability and Transcription Elongation Impairment in Human Cells Depleted of THO/TREX

**DOI:** 10.1371/journal.pgen.1002386

**Published:** 2011-12-01

**Authors:** María S. Domínguez-Sánchez, Sonia Barroso, Belén Gómez-González, Rosa Luna, Andrés Aguilera

**Affiliations:** Centro Andaluz de Biología Molecular y Medicina Regenerativa (CABIMER), Universidad de Sevilla – Consejo Superior de Investigaciones Científicas (CSIC), Sevilla, Spain; The Hospital for Sick Children and University of Toronto, Canada

## Abstract

THO/TREX connects transcription with genome integrity in yeast, but a role of mammalian THO in these processes is uncertain, which suggests a differential implication of mRNP biogenesis factors in genome integrity in yeast and humans. We show that human THO depletion impairs transcription elongation and mRNA export and increases instability associated with DNA breaks, leading to hyper-recombination and γH2AX and 53BP1 foci accumulation. This is accompanied by replication alteration as determined by DNA combing. Genome instability is R-loop–dependent, as deduced from the ability of the AID enzyme to increase DNA damage and of RNaseH to reduce it, or from the enhancement of R-loop–dependent class-switching caused by THOC1-depletion in CH12 murine cells. Therefore, mammalian THO prevents R-loop formation and has a role in genome dynamics and function consistent with an evolutionary conservation of the functional connection between these mRNP biogenesis factors and genome integrity that had not been anticipated.

## Author Summary

THO/TREX is an eukaryotic conserved complex, first identified in budding yeast, that acts at the interface between transcription and mRNP (ribonucleoprotein) export. In yeast, THO mutants show gene expression defects and a transcription-associated recombination phenotype. Despite the structural conservation of THO/TREX, it is unclear whether the functional relevance is the same in mammals, in which several reports have identified a role of THO/TREX separated from transcription. We have asked whether mammalian THO/TREX function is connected to transcription and whether this function is required to prevent R-loop formation and to maintain genome integrity. Our study reveals that depletion of human THO subunits, in particular THOC1/hHPR1, reduces transcription elongation, affects mRNA export, and increases genome instability associated with the accumulation of DNA breaks. This genome instability is R-loop–dependent and is accompanied by an alteration of global replication patterns and an increase in recombination. We conclude that human THO/TREX prevents the formation of R-loops that can compromise genome integrity. This work, therefore, provides experimental evidence for a role of mRNP biogenesis factors and R loops in genome integrity in humans.

## Introduction

Transcription is a central cellular process occurring in the nucleus of eukaryotic cells in coordination with other nuclear processes. During transcription, the nascent pre-mRNA associates with mRNA-binding proteins and undergoes a series of processing steps, resulting in export-competent mRNA ribonucleoprotein complexes (mRNP) that are transported into the cytoplasm. The different steps of mRNP biogenesis are coupled to each other via an extensive network of physical and functional interactions [1], [2].

THO is a structural and functional unit identified first in budding yeast that is composed of four-protein (Hpr1, Tho2, Mft1, Thp2) and is associated with Tex1 and the mRNA export factors Sub2 and Yra1 forming a larger complex termed TREX [3], [4]. THO mutations lead to gene expression defects that are particularly evident for long and GC-rich DNA sequences [3], as well as for repeat-containing genes [5]. Such defects are the consequence of an impairment in transcription elongation as determined both *in vivo* and *in vitro* [3], [6], [7]. THO mutants show a hyper-recombination phenotype that is associated with transcription and is dependent on the nascent RNA molecule and on the co-transcriptional formation of RNA-DNA hybrids (R-loops) [8], [9]. In the current view, yeast THO would participate in the co-transcriptional formation of export-competent mRNP during transcription elongation by controlling the assembly of heterogeneous nuclear ribonucleoproteins (hnRNPs) onto the mRNA [10].

THO/TREX is conserved in all eukaryotes, and has been purified in *Drosophila* and human cells [4], [11], [12]. The human TREX (hTREX) complex is composed of the multimeric THO (hTHO) complex, containing hTHO2/THOC2, hHpr1/THOC1, fSAP79/THOC5, fSAP35/THOC6, fSAP24/THOC7 and hTex1/THOC3, the DEAD-box RNA helicase Sub2/UAP56 and the mRNA export adaptor protein Yra1/Aly/THOC4 [12]. Interestingly, it is associated with the spliceosome proteins and with spliced RNA, the latter interaction being independent of transcription, which raises the question of whether or not the involvement of THO/TREX in transcription is general from yeast to humans [12]. There is also evidence for transcription-dependent recruitment of THO to chromatin in both *Drosophila* and human cells [13], [14], but whether or not this is due to the known co-transcriptional function of the splicing machinery is still an open question. In this sense, hTREX has been shown to be recruited to the 5′ cap site of the mRNA via an interaction between ALY and the cap-binding complex CBC during splicing, ensuring mRNA export to the cytoplasm in a 5′ to 3′ direction [13]. ALY is a well-conserved RNA-binding protein that physically interacts with the conserved mRNA export Mex67/Tap/NXF1 allowing the mRNA-protein complex to be exported through the nuclear pore [15].

Despite the conservation of THO/TREX it is unclear whether the functional relevance is the same in all eukaryotes, which is important to know the degree of coupling between transcription and RNA export in higher eukaryotes. Thus, for example, in Drosophila the THO complex, is not essential for bulk poly(A)+ RNA export, whereas this is the case for UAP56 [16]–[19]. Whether human THO depletion impairs transcription elongation, mRNP biogenesis or RNA export or has genome-wide or transcript-specific effect is still an open question [11], [12], [19]–[22].

A distinctive phenotype of yeast THO mutants is their hyper-recombination phenotype associated with transcription, which is shared by other mRNP biogenesis/export factors from yeast to humans [23]–[26]. It has long been established that transcription enhances homologous recombination from bacteria to mammalian cells, a phenomenon termed TAR (transcription-associated recombination) [27]. However, whereas TAR in yeast THO mutants is dependent on the nascent mRNA molecule and is associated with R-loop formation, this has not been shown for human THO depletion.

In this work the effect of human THO depletion has been investigated on cell proliferation, transcription elongation and genome stability. Our study reveals that depletion of human THO subunits, in particular THOC1/hHPR1, reduces transcription elongation and RNA export, as determined by nuclear mRNA accumulation. hTHO depletion in different cell lines increases instability associated with the accumulation of DNA breaks, such instability being R-loop-dependent. Consistently, R-loop-dependent class-switching recombination is enhanced by THOC1 depletion in murine CH12 cells. Altogether, this work provides evidence for a functional role of THO in transcription and RNA-dependent genome instability, supporting a function of human THO/TREX in chromatin dynamics and function. These results indicate that the connection of transcription and mRNP biogenesis with genome instability is more functionally conserved from yeast to humans than previously anticipated.

## Results

### Gene expression defects in THO/TREX knockdown cells

**Figure 1:**
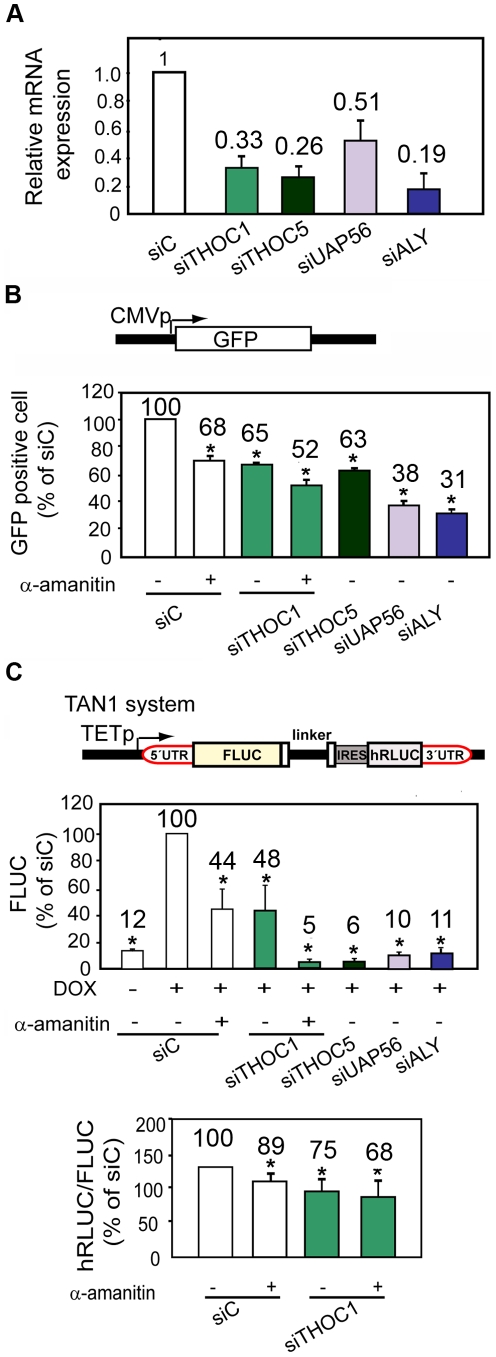
THO/TREX depletion impairs transcription elongation. A) Relative expression of THO/TREX components. mRNA levels were measured by RT-qPCR 96 h after siRNA depletion. B) GFP expression determined by FACS analysis after 96 h of depletion with siRNAs (hHpr1/THOC1, THOC5, UAP56 and ALY), performed 24 h after transfection with the pmaxGFP vector containing CMVp::GFP. α-amanitin (2 µg/ml) was used as a positive control of transcription inhibition. A scheme of the pmaxGFP reporter carrying a CMVp::GFP fusion is shown on top. C) Transcription elongation determined with TAN system. Scheme of the tandem reporter system TAN1 used for transcription elongation. A tetracycline-regulated promoter (TETp) drives transcription through the FLUC and hRLUC tandem reporters. An internal ribosome entry sequence (IRES) enhances translation of the uncapped hRLUC expression fragment by replacing the requirement for the 5′ cap and untranslated region (5′ UTR). FLUC expression determined by luminometer analysis with TAN1 is shown. hRLUC∶FLUC activity ratios are plotted for the indicated 96 h siRNA depleted cells. For this, 72 h after siRNA transfection, cells were transfected with TAN1, transcription was activated with doxycycline, and 24 hours later, cells were harvested. Results are expressed as a percentage of the siRNA control (siC). Average and standard error from three independent experiments are shown. When the P value of the difference with the siC control calculated with the Student's *t* test is <0.05, it is indicated with an asterisk (*).

To assay the implication of hTHO/TREX in gene expression, the effect of gene silencing of different THO/TREX components by RNA interference was investigated. HeLa cells were transfected with siRNAs against hHpr1/THOC1, THOC5, UAP56 and ALY and total RNA was analyzed by RT-qPCR. After transfection THOC1, THOC5, UAP56 and ALY mRNA levels were reduced to 33%, 26%, 51% and 19%, respectively, compared to the levels of the cells transfected with the siC control (Figure 1A).

Next we asked whether this depletion had a significant effect on gene expression of a reporter gene. For this purpose 72 h siRNA depleted HeLa cells were newly transfected with plasmid *pmax* containing a GFP cDNA. GFP gene expression was analyzed 24 h later by flow cytometry. Figure 1B shows the levels of GFP expression in the siRNA-transfected HeLa cells. The relative percentage of GFP positive cells was calculated for each siRNA transfected cell line. A reduction of approximately 40% of GFP expression was observed in cells transfected with siTHOC1 and siTHOC5, a similar percentage (about 50% of GFP) detected for cells treated with the transcription inhibitor α-amanitin, used as positive control in the experiments. The most drastic change, however, was observed in cells transfected with siUAP56 and siALY with a reduction close to 70% in the percentage of GFP positive cells. A reduction in the expression of a constitutive endogenous HPRT gene by RT-qPCR was also detected (Figure S1A). Altogether these results support that hTHO/hTREX depletion in human cells causes transcription defects.

### Transcription elongation impairment in THO/TREX-depleted cells

To investigate whether the conserved human complex has a role in transcription elongation we used a tandem system (TAN1) to measure transcription elongation in cells depleted of THOC1 and other mRNP factors. This system consists of a single transcriptional unit covering two reporter genes, FLUC and hRLUC, under the control of the doxycycline inducible *tet* promoter [28] (Figure 1C). As the two reporter sequences are transcribed from a unique promoter, the ratio of expression of the downstream reporter *versus* the upstream reporter provides a measure of the relative rate of successful elongation through the intervening sequence. First, we evaluated the expression of the reporter FLUC in cells transfected with siTHOC1 *versus* the siC control and α-amanitin treated cells. A reduction of 50% of the reporter expression was observed in α-amanitin treated cells and a comparable but slightly lower reduction was detected with siTHOC1 (Figure 1C, upper panel). Interestingly, when the siTHOC1 transfected cells were treated with α-amanitin, a synergistic effect was observed (90% of reduction in FLUC expression). The ratio of hRLUC to FLUC activities in THOC1-depleted cells was reduced and in the presence of α-amanitin a synergistic effect was observed again (Figure 1C, lower panel), indicative of a role of THOC1 in transcription elongation. A strong reduction of FLUC activity was observed with siTHOC5, siUAP56 and siALY, consistent with a relevant role of these three subunits in transcription. However, due to these low FLUC values, it was not possible to obtain reliable hRLUC/FLUC ratios in these cases. To confirm that the effect observed in transcription elongation deduced from the hRLUC and FLUC activities, we determined the levels of transcripts containing each segment by qRT-PCR. The results clearly indicate that the hRLUC∶FLUC ratios of mRNA levels was significantly reduced in the cell lines depleted of the 4 subunits analyzed, THOC1, THOC5, UAP56 and ALY (Figure S1B), confirming a general role of THO/TREX in transcription elongation in human cells.

### Increased DNA breaks and recombination in THO/TREX-depleted cells

**Figure 2:**
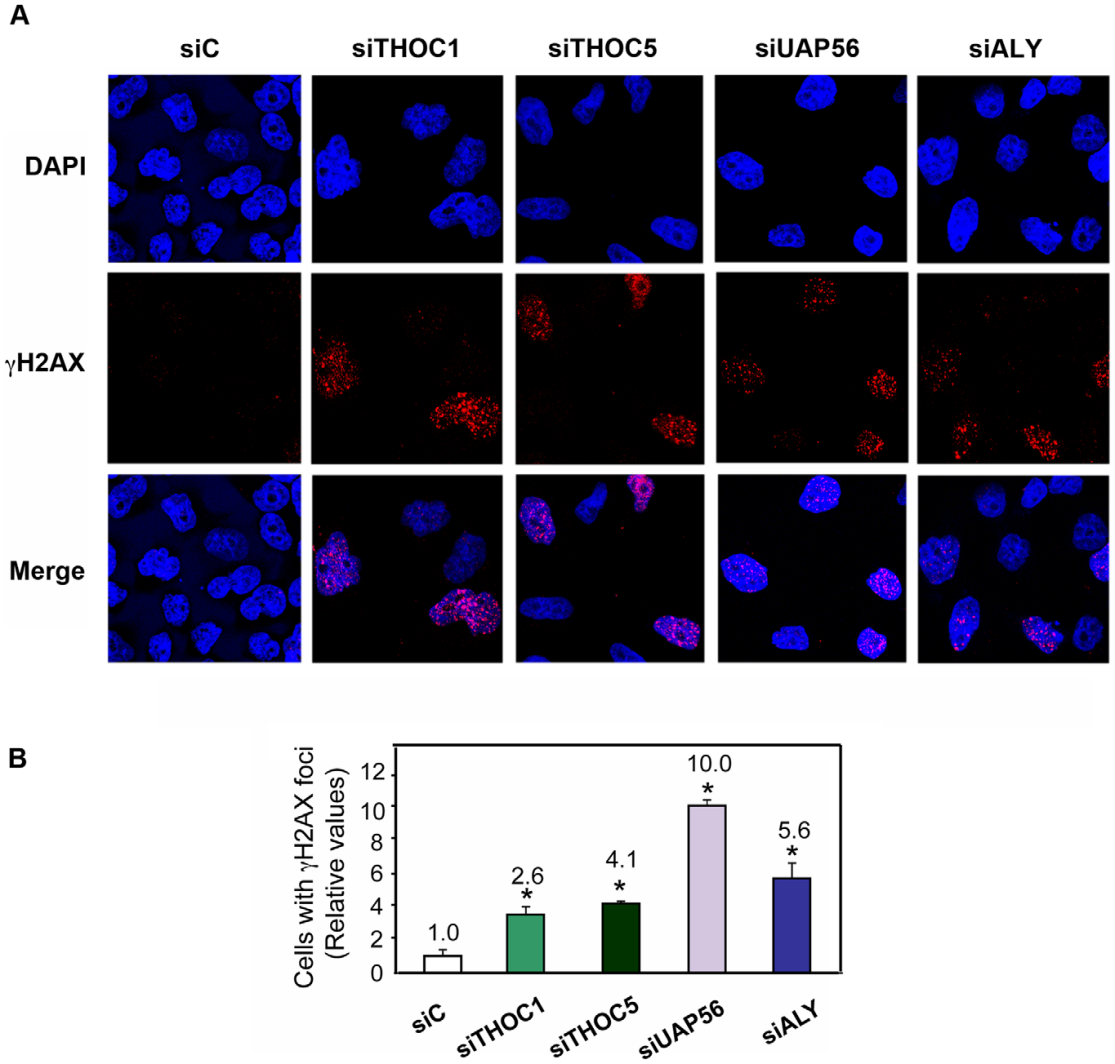
THO/TREX depletion increases cellular DNA damage response. A) Immunofluorescence of γH2AX after transfection with the indicated siRNAs. Time-course experiments were performed. The time point where the maximum γH2AX foci containing cells were observed for each depletion is shown (48 h for siTHOC1 and siTHOC5, and 72 h for siUAP56 and siALY). Nuclei were stained with DAPI. B) Quantification of γH2AX after siRNA depletion is shown. . Average and standard error from three independent experiments are shown (more than 100 cells were analyzed per siRNA transfection). When the P value of the difference with the siC control calculated with the Mann & Whitney test is <0.05, it is indicated with an asterisk (*).

Since homologous recombination in mitotically dividing cells is the consequence of the repair of DNA breaks, the accumulation of DNA breaks was determined in THO/TREX-depleted cells by measuring the accumulation of γH2AX foci, one of the first components of the DNA damage response [29]. γH2AX *in situ* localization in HeLa cells transfected with siRNAs against hHpr1/THOC1, THOC5, UAP56 and ALY show clearly that transient depletion of these factors causes an accumulation of DNA damage, as deduced from the 2.6–10-fold increase in the number of cells containing γH2AX foci (Figure 2). Experiments were also performed in HeLa cell lines in which THO/TREX subunits were depleted by shRNAs. We first showed that shTHOC1, shUAP56 and shALY reduced the levels of THOC1, UAP56 and ALY mRNA to 30–40% of the levels of the cells transfected with the shTM blank control, as determined by RT-qPCR after 48 h of transfection (Figure S2A). In this case, in addition to γH2AX foci we analyzed the levels of the 53BP1 DNA-damage checkpoint protein [30]. The levels of γH2AX and 53BP1 foci were increased (Figure S2B and S2C), although to a lesser extent as in the siRNA-depleted cells, likely due to an earlier and more efficient protein depletion with siRNAs transfection.

**Figure 3:**
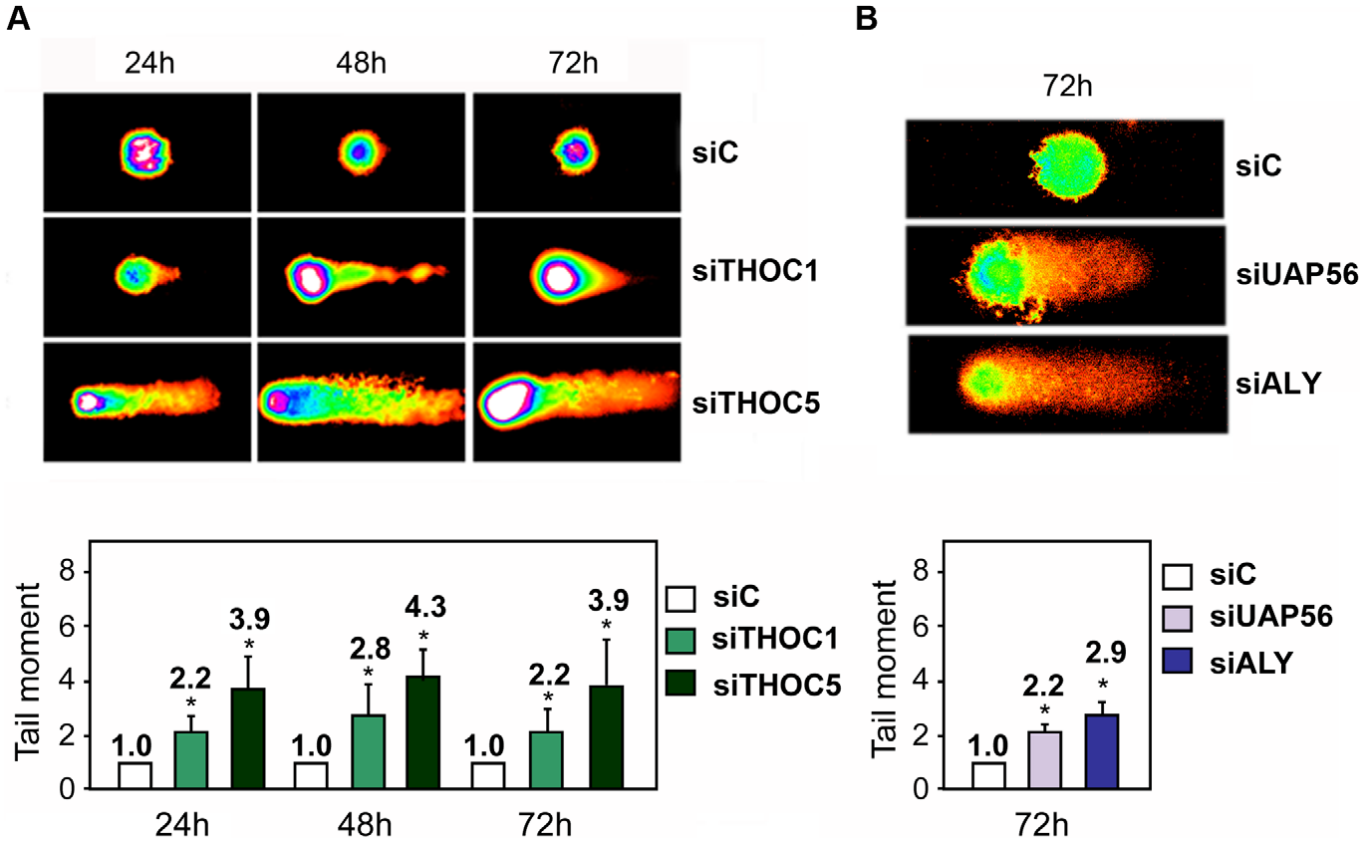
THO/TREX depletion leads to an increase in DNA breaks. A) Quantification of DNA breaks at sequential time points after transfection with siTHOC1 and siTHOC5 siRNAs assessed by the alkaline comet assay. B) Comet assay 72 h after transfection with siUAP56 and siALY siRNAs. At least 50 cells were counted per group to calculate the median of the tail moment. Average and standard error from three independent experiments are shown. When the P value of the difference with the siC control calculated with the Mann & Whitney test is <0.05, it is indicated with an asterisk (*).

Next, DNA damage was directly assessed by single-cell electrophoresis (Comet assay) by which, following DNA unwinding under alkaline conditions, broken DNA fragments (damaged DNA) migrate away from the nucleus (see Materials and Methods). First, we performed comet assay at different times in HeLa cells transfected with siTHOC1 and siTHOC5. As can be seen in Figure 3A, THO depleted cells show a significant increase in the tail moment. Similar results were obtained in siTHOC1 and siTHOC5-depleted MRC5 cells (Figure S3), a fibroblast cell line derived from normal lung tissue, indicating that THO depletion leads to an accumulation of DNA breaks in both normal and tumoral cell lines. Finally, to assay whether this accumulation occurred in cells depleted of other THO/TREX subunits, we performed the same experiments in HeLa cells transfected with siUAP56 and siALY siRNAs. As can be seen in Figure 3B, after 72 h of siRNA transfection, the cells showed a significant increase in DNA breaks as determined by the Comet assay.

### Stable cell lines for inducible shTHOC1 show transcription and mRNA export defects

**Figure 4:**
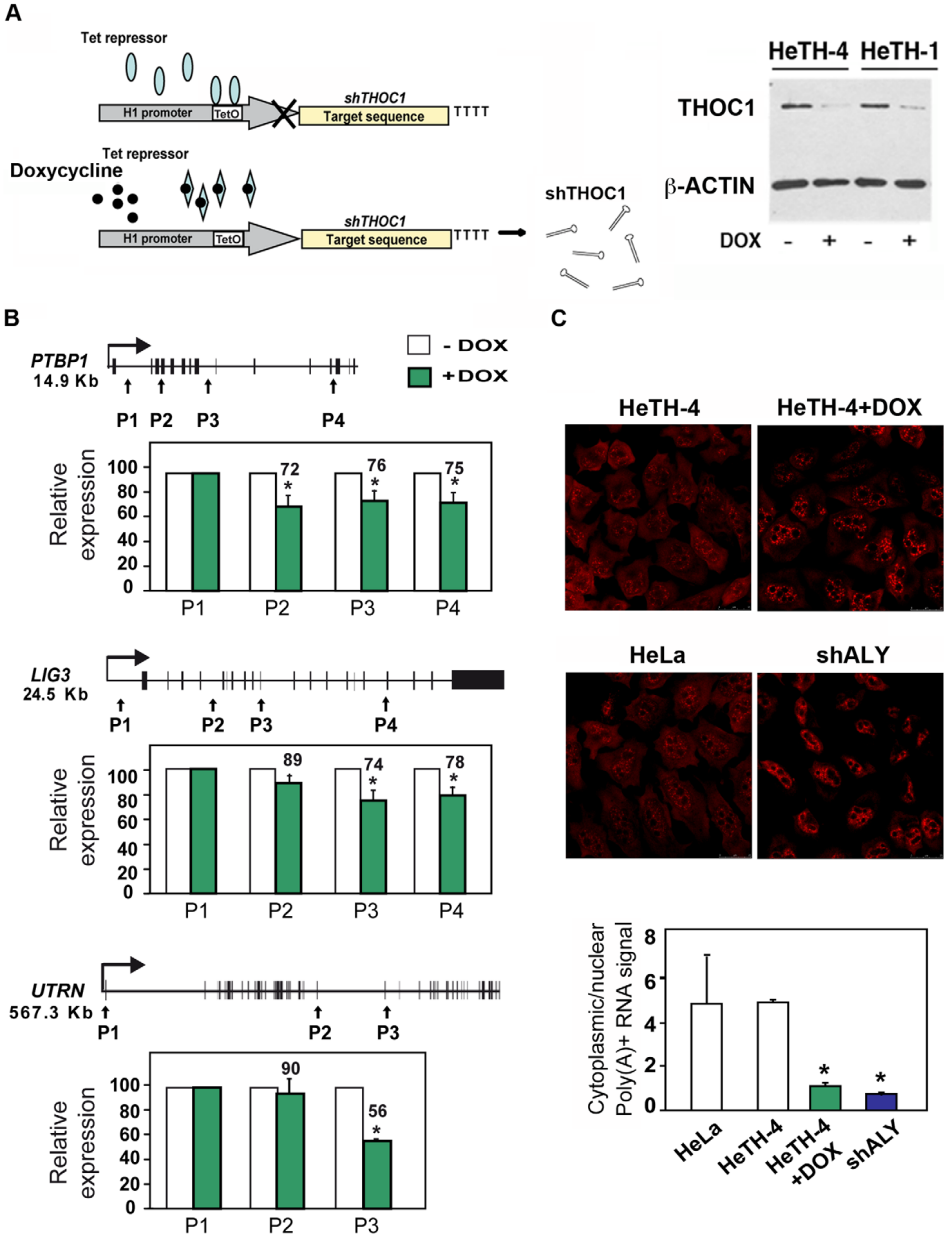
Gene expression defects in stable cell lines depleted of THOC1. A) Immunoblot showing THOC1 expression in HeLa stable cell lines with an inducible shTHOC1 (HeTH cells) with (+DOX) or without doxycycline (−DOX). A scheme of the system used to induce THOC1 shRNA expression is shown. B) Effect of THOC1 depletion in transcription elongation of endogenous genes (PTBP1, LIG3 and UTRN) as determined by RT-qPCR. The relative amount of nascent mRNA in HeTH-4 cells is plotted. C) Nucleocytoplasmic polyA+ RNA distribution in HeLa-derived HeTH-4 cells in which THOC1 depletion was induced with doxycycline and HeLa cells transiently transfected with the indicated shRNAs. More than 50 cells per group were subjected to *in situ* hybridization with Cy3-oligo dT_50_ probe. Scale bar refers to 25 µm. Ratio of cytoplasmic and nuclear signals as quantified in each knockdown cell is represented below. Average and standard error of 3 independent experiments are shown. When the P value of the difference with the siC control calculated with the Student's *t* test (for results shown in B) or the Annova-Newman and Keuls (for results shown in C) is statistically significant it is indicated with an asterisk (* for P<0.05).

Once demonstrated that THO/TREX depletion has an impact on gene expression, regardless of the subunit depleted, we decided to continue the analysis with the THOC1 conserved subunit as a representative THO subunit. For this reason, we first constructed stable HeLa cells lines for the depletion of THOC1. HeLa cells were stably transfected with an inducible shRNA for THOC1 (see Materials and Methods). Stable integration of the inducible THOC1 shRNA vector allowed the rapid production of siRNAs upon doxycycline induction. Among 4 stable clones obtained, HeTH-1 and HeTH-4 were chosen, showing about 50% reduction on THOC1 mRNA levels as determined by RT-qPCR (data not shown) and an efficient knock-down of the THOC1 protein as determined by Western analysis (Figure 4A). As expected from previous works [31], the growth rate of these stable cell lines was significantly reduced (Figure S4).

The impact of THOC1 depletion on transcription of endogenous genes was analyzed in HeTH-4 cells (+DOX) (+doxycycline) compared to control cells HeTH-4 cells (-DOX) (Figure 4B). These kinds of analyses have been previously used to study the role in transcription elongation of splicing factors [32]. RT-qPCR on DNase I-treated total RNA was performed using primer pairs covering different regions of three different-sized genes: *PTBP1* (polypyrimidine tract binding protein 1), *LIG3* (ligase III, DNA, ATP-dependent) and *UTRN* (utrophin). A reduction in the amount of mRNA at the 3′ proximal regions *versus* the 5′ ones were observed for the three endogenous genes analyzed upon doxycycline addition (Figure 4B). A similar reduction was observed when we added the transcription inhibitor α-amanitin (Figure S5). These results suggest that THOC1 depletion has a negative effect on transcription elongation in human cells.

We performed a global analysis of transcription to see whether the effect was general and to explore whether an effect on a specific transcription or mRNP biogenesis factor could indirectly explain the previous results. Comparison of the gene expression profiles between THOC1-depleted cells (HeTH-4 +DOX) cells with mock-treated controls (HeTH-4 –DOX) revealed that out of 28869 genes, 94 were down-regulated (32 well annotated genes and 62 non-coding RNAs (ncRNA)) and 140 up-regulated (36 well annotated genes and 104 ncRNAs), taking as a threshold set at 1.5-fold difference. (Table S2). Gene-GO term enrichment analysis does not show any relevant GO term associated with the list of gene deregulated. These data suggest that the effect of THOC1 depletion on transcription could be direct and not mediated by the altered expression of other genes.

Yeast THO mutants have a global poly(A)+ mRNA export defect [4], whereas in Drosophila THO is required for nuclear export of heat-shock mRNAs but it seems dispensable for nuclear export of total mRNA [11]. However, in human cells ambiguous data about the role of the THO complex in export of bulk poly(A)+RNA have been reported [20], [21]. To explore whether THOC1 is required for nuclear export of bulk poly(A)+ RNA in human cells *in situ* hybridization assays were performed with a fluorescently labeled oligo(dT) probe in HeTH-4 cells (+DOX) (Figure 4C). A series of optical sections through the entire cell was analyzed by confocal microscopy and the fluorescence signal in the cytoplasmic and nuclei was compared. The analysis revealed that whereas the non-induced shTHOC1 control cells showed uniform poly(A)+ distribution in the cell, similar to that of untransfected HeLa cells, poly(A)+RNA accumulated in a non-uniform manner in HeTH-4 cells (+DOX). The pattern was similar to that observed in HeLa cells transfected with a plasmid bearing a shRNA specific of ALY, used as positive control (Figure 4C). Accordingly, a significant reduction in the cytoplasmic-nuclear (C/N) ratio was observed with respect to that of HeLa control cells. Altogether these results suggest a general role of THOC1 in transcription and RNA export.

### Stable cell lines for inducible shTHOC1 show an increase in DNA breaks and recombination

**Figure 5:**
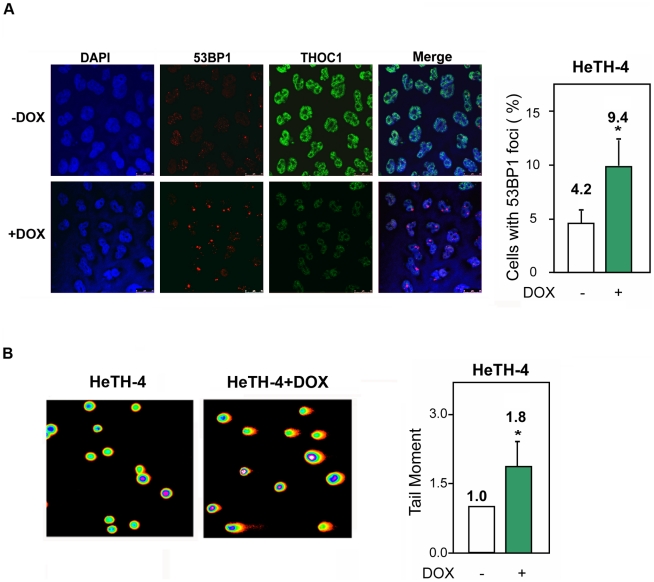
Genome instability in stable cell lines depleted of THOC1. A) 53BP1 foci formation in HeTH-4 cells (−DOX) and HeTH-4 cells depleted of THOC1 (+DOX). Immunofluorescence of THOC1 and 53BP1 are depicted. Nuclei were stained with DAPI. The percentage of cells with 53BP1 foci in the presence (−DOX) or absence (+DOX) of THOC1 is plotted. B) DNA breaks measured by the Comet assay in HeTH-4 cells with or without doxycycline. The graph shows the increase in the tail moment as indicative of DNA breaks occurring after THOC1 depletion. Average and SE from three independent experiments are shown (number of cells analyzed as in Figure 2 and Figure 3). When the P value of the difference with the control calculated with the Mann & Whitney test is <0.05, it is indicated with an asterisk (*).

To further investigate how THO depletion induces DNA damage, we focused our efforts on THOC1-depletion using as a tool the stable HeTH-4 cell line expressing the inducible shTHOC1. First, we confirmed that the accumulation of 53BP1 foci after depletion of this factor also take place in the stable cell line. The strong reduction of THOC1 (+DOX) was accompanied by a 2-fold increase in 53BP1 foci with respect to the control (−DOX) (Figure 5A).

Next, DNA damage was assessed by single-cell electrophoresis (Comet assay) by which, following DNA unwinding under alkaline conditions, broken DNA fragments (damaged DNA) migrate away from the nucleus (see Materials and Methods). A two-fold increase in the tail moment in THOC1-depleted HeTH-4 cells (+DOX) demonstrates the accumulation of DNA breaks (Figure 5B).

**Figure 6:**
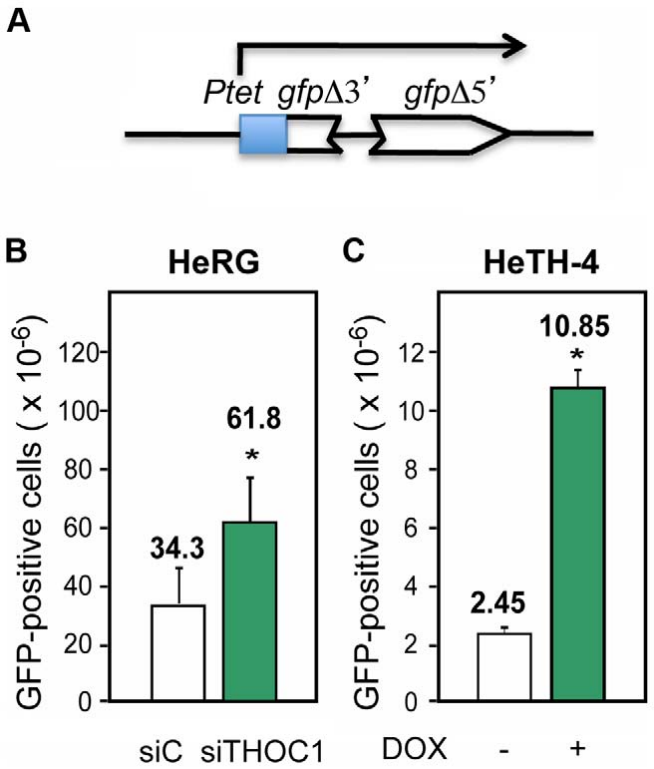
Recombination is increased after THOC1 depletion. A) Scheme of the pIREC direct-repeat recombination construct used to generate stable HeLa cell lines. B) Recombination analysis using the HeRG stable cell line depleted of THOC1 by siRNA. C) Recombination analysis in the HeTH-4 stable cell lines (−DOX and +DOX) transfected with the plasmid pIREC-direct repeat containing vector. The recombination frequency was measured by FACs analysis as GFP positive cells, 96 h after transfection (B and C). Average and SE from three independent experiments are shown. When the P value of the difference with the siC control calculated with the Mann & Whitney test is <0.05, it is indicated with an asterisk (*).

Finally, to assay whether the increase in DNA breaks in THO/TREX depleted cells resulted in an increase in recombination, we designed and constructed a direct-repeat recombination construct, pIREC (Figure 6A). It consists of two GFP truncated repeats sharing 200-bp of homology and placed under the control of the doxycycline inducible *tet* promoter that were stably integrated into the genome (HeRG) (see Materials and Methods). Recombinants in this assay could be detected by FACS analyses as GFP positive cells. As can be seen in Figure 6 in both the HeRG stable cell line transfected with siRNA to deplete THOC1 (Figure 6B) and in HeTH4 cells (+DOX) transfected with the pIREC system (Figure 6C), the spontaneous recombination frequency increased with respect to their respective controls, consistent with an increase in DNA breaks and subsequent recombination events taking place upon THO depletion.

### Genome instability in THOC1 depleted cells is R-loop–dependent

**Figure 7:**
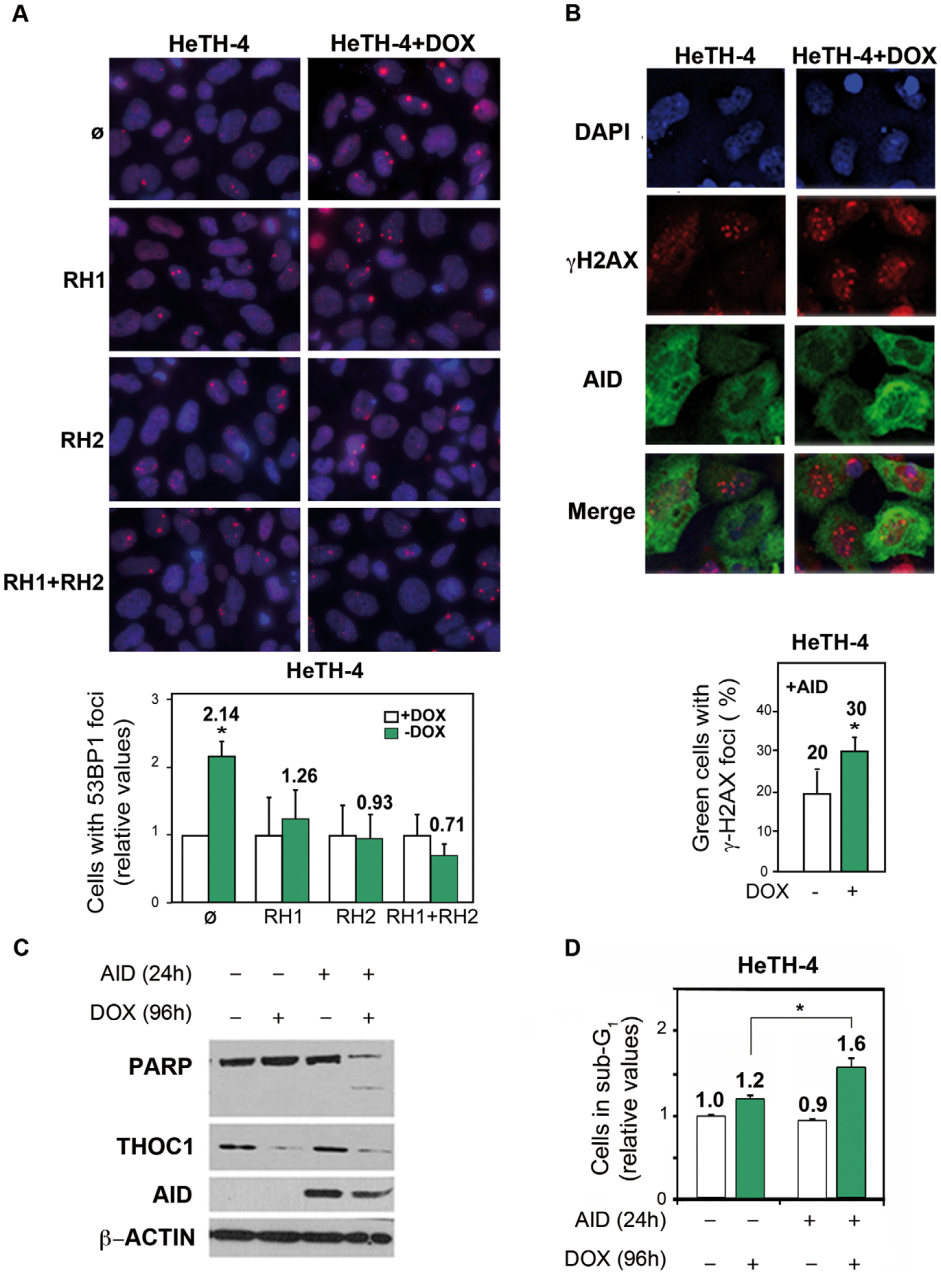
Genome instability in THOC1 is mediated by R-loop formation. A) Suppression of the increase in the number of cells with 53BP1 foci in HeTH-4 cells (+DOX) by RNaseH overexpression. Cells were transfected with pcDNA3 (ø), pcDNA3-RNaseH1 (RH1) and/or pcDNA3-RNaseH2 (RH2). Merge images of DAPI nuclei stained and 53BP1 immunofluorescence are shown. B) Immunofluorescence of γH2AX and AID pictures are depicted. Nuclei were stained with DAPI. Graph shows the increase in the percentage of cells with foci in the condition of THOC1 depletion and AID expression. Average and SE from three independent experiments are shown (more than 100 cells were analysed per group). C) Analysis of the effect of AID expression on apoptosis in THOC1-depleted cells as determined by Immunoblot of HeTH-4 cell extracts using anti-PARP antibody. D) Analysis of the effect of AID on apoptosis in THOC1-depleted cells as determined by FACS analysis of cell displaying subG_1_-DNA content. Average and SE from three independent experiments are shown. When the P value of the difference with the control calculated with the Mann & Whitney test is <0.05, it is indicated with an asterisk (*).

Next we wondered whether DNA breaks in THO-depleted human cells were a consequence of R-loop formation. THOC1-depleted HeTH-4 cells (+DOX) were transfected with vectors expressing RNaseH (*RNH1* and/or *RNH2*), which degrades the RNA strand of DNA-RNA hybrids, and the 53BP1 foci formation was measured. Overexpression of *RNH1*, *RNH2* or both reduced 53BP1 foci to values close to control levels, consistent with R-loop formation, although we can not rule out other sources of genome instability (Figure 7A). To further confirm this result, we tested whether human AID, a cytidine deaminase which works in single-stranded DNA as those formed in R-loops, increased DNA breaks in THOC1-depleted cells, an assay that has been used successfully in yeast [9]. As shown in Figure 7B AID expression in HeTH-4 cells (+DOX) increased the percentage of cells containing γH2AX foci 1.8-fold. Indeed, it is worth noting that after AID overexpression we detected PARP degradation in THOC1-depleted cells as determined by western (Figure 7C), suggesting that under these conditions a cell death program could be induced. According to these data an increase in the number of apoptotic cells, as measured by sub-G_1_ DNA content, was detected (Figure 7D).

### Class-switching enhancement in THOC1-depleted murine CH12 cells

**Figure 8:**
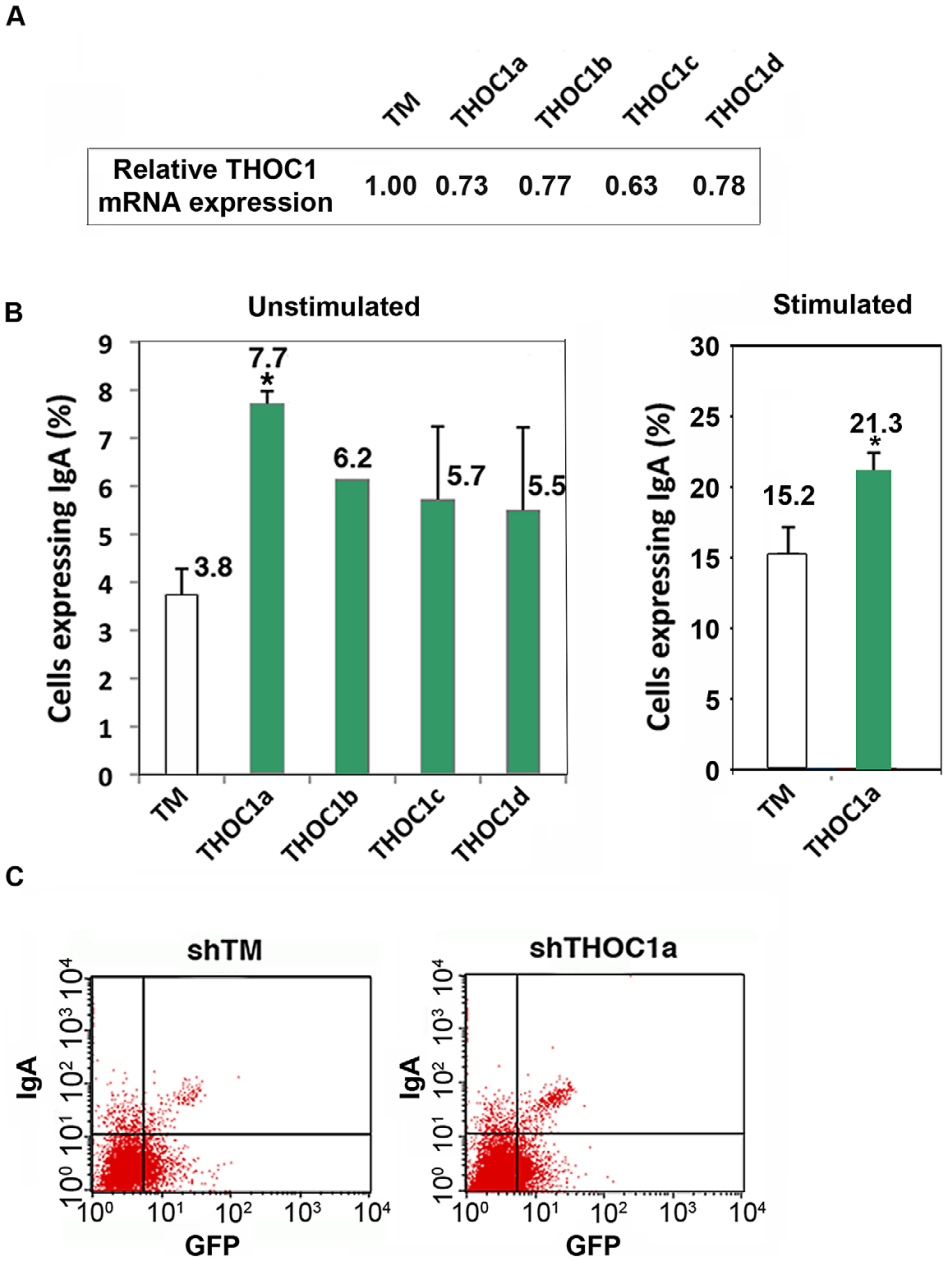
Depletion of THOC1 in CH12 murine B cell line enhances class switch recombination. CH12 cells were transfected with a pSUPER vector containing GFP and the indicated shRNA (lower case letters indicate shRNAs against different gene exons). A) Relative THOC1 mRNA levels. B) IgA expression was measured 72 h after transfection in GFP positive cells by FACs in unstimulated and stimulated cells (treated with cytokines for 12 h, as detailed in Material and Methods). When the P value of the difference with the control calculated with the Mann & Whitney test is <0.05, it is indicated with an asterisk (*). C) A representative graph of the FACs experiments.

Class switching is a natural phenomenon of recombination that is dependent on R-loop formation at the switch S regions of Immunoglobulin genes [33]. If THO-depletion facilitates R-loop formation in mammalian cells, we reasoned that in B cells AID would enhance its spectrum of action. To test this possibility class switching was assayed in murine CH12 cells derived from B cell lymphoma that were depleted of different subunits of murine THO by siRNA against different exons of THOC1. As can be seen in Figure 8B, there is a clear and consistent increase of class switching, in both unstimulated and stimulated cells, as determined by IgM to IgA conversion measured by FACS analyses (see Materials and Methods). The enhancement of the basal level of class switching detected in unstimulated cells can be explained by the direct action of AID on the S region, as it has been previously reported that AID induction augments class switching of unstimulated CH12 cells, which are known to express germline transcripts even without stimulation [34], [35]. In agreement with these data we detected AID and Iμ transcripts in unstimulated cells as determined by RT-PCR (Figure S6). The increase in class-switching in CH12 cells after THOC1 depletion support our hypothesis that THO-depletion could enhance the ability of mammalian cells to form recombinogenic R-loops.

### Replication fork progression alterations in THOC1-depleted cells

**Figure 9:**
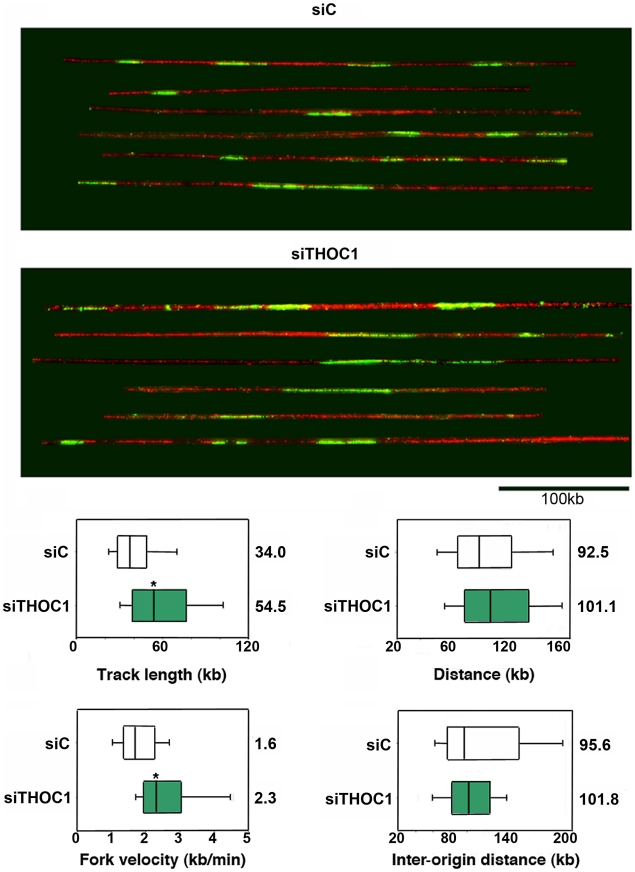
Combing assay showing that replication fork progression is altered in cells depleted of THOC1. Single-molecule analysis of DNA replication. siC (control) and siTHOC1 transfected HeLa cells were pulse-labeled for 20 min with CldU and fibres were stretched by DNA combing. Red: DNA, Green: CldU. Bar: 100 kb. Distribution of CldU tracks length in HeLa cells. Box: 25–75 percentile range. Whiskers: 10–90 percentile range. Medians are indicated in kb. Distribution of centre-to-centre distances between CldU tracks, replication fork velocity and inter-origin distance in HeLa cells transfected with siC and siTHOC1 siRNAs are shown. When the P value of the difference with the siC control calculated with the Median test is <0.05, it is indicated with an asterisk (*).

One main function of recombination is to repair the DNA breaks that occur spontaneously as a consequence of DNA replication stalling or collapse. We asked whether the breaks and hyper-recombination of THOC1-depleted cells were accompanied by replication defects. Therefore, HeLa cells were transfected with siTHOC1 and siC and pulse-labeled with CldU (Chlorodeoxyuridine) to monitor replication by DNA combing. This analysis revealed that CldU tracks, which visualize newly replicated regions, are longer in siTHOC1 cells (54.5 kb) than in the siC control cells (34.0 kb) (Figure 9), suggesting that replication was 30% faster in THOC1-depleted cells. Similar frequency of replication initiation, as inferred from the distance between the centers of two CldU tracks, were observed in THOC1-depleted siTHOC1 (101.1 kb) and siC control cells (92.5 kb) (Figure 9). To measure replication elongation, cells were pulse-labeled with IdU and CIdU and the distance covered by individual forks during the pulse was determined. Results showed that replication forks travel at an apparent faster speed in THOC1-depleted siTHOC1 (2.3 kb/min) than in siC (1.6 kb/min) cells (Figure 9). Similar results were obtained with the stable cell line HeTH-4 (Figure S7). Instead, no clear differences were observed in the frequency of origin firing, as the inter origin distance in siTHOC1 was similar to that of control cells (Figure 9). It seems therefore clear that THOC1 depletion alters the progression of replication fork.

## Discussion

In this study we provide evidence that in human cells the THO/TREX complex has a role in mRNP biogenesis that connects transcription elongation, mRNA export and genetic instability. Reducing the expression of human THO/TREX components by RNA interference experiments results not only in a reduction of gene expression and mRNA export, but also in an impairment of transcription elongation. Moreover, we show that human THO depletion increases instability associated with DNA breaks, as determined by hyper-recombination and γH2AX and 53BP1 foci accumulation. Notably, such instability is dependent on R-loop formation, as determined by different *in vivo* approaches, and correlates with an alteration of global replication patterns as determined by DNA combing. Altogether these data suggest that human THO is a key player for mRNP formation and genome integrity that connects transcription elongation with genome dynamics and reveals that the connection of transcription and mRNP biogenesis with genome instability is more conserved than previously anticipated.

### Human THO/TREX functions at the interface of transcription elongation and mRNA export

Our analyses of a tandem transcription reporter construct, and nascent mRNAs from different endogenous genes by RT-qPCR (Figure 1, Figure S1 and Figure 4) indicate that THO has a role in transcription elongation. The impact of THO depletion seems to be general and direct. The microarray analysis does not identify a significant reduction of expression of genes involved in mRNP biogenesis that could explain the results (Table S2). Different results have been reported for THO/TREX co-precipitation with the transcription apparatus [12], [31], [36], but our data suggests that THO/TREX have a functional role coupled to transcription elongation. Consistently, an early recruitment of THO to the 5′ end of mRNAs has been shown in a splicing- and cap-dependent manner [13]. This recruitment requires the cap-binding subunit CBP80, which interacts with the ALY/REF subunit of human TREX, and could explain that mRNA export takes place through the nuclear pore in a 5′ to 3′ direction.

RNA interference and biochemical studies in metazoans and genetic analyses in yeast indicate that the conserved THO/TREX complex functions in mRNA export [15], [20] (Figure 4C; [21]). However, in *Drosophila* the nuclear export of only a subset of mRNAs is affected by depletion of the THO subunits, which depends on the subunit depleted [11], [19]. In light of these results, the existence of various nuclear mRNA export pathways in multicellular eukaryotes has been suggested, which may be dictated by different adaptor RNA binding proteins. Consistently, it has been shown that THOC5, a subunit of the metazoan THO complex with no apparent orthologue in yeast, is not required for bulk mRNA export. However, it interacts with TAP-p15 and ALY, and functions in the export of specific mRNAs such as HSP70 [20].

The number of factors working at the interface transcription elongation and mRNA export reveals an increasing importance of the tight association between transcription and RNA biogenesis steps. Thus, Drosophila THO and ENY2/Sus1, a component of the histone-acetyltransferase complex SAGA/TFTC involved in transcription activation, interacts with the THSC/TREX-2 complex, required for mRNA export [14]. Also the human hnRNP CIP29 protein, the ortholog of yeast Tho1 hnRNP functionally related with THO, has been shown to be recruited to THO and to participate in mRNA export [37]. Spt6, a transcription elongation factor and histone H3 chaperone, binds to the Ser2P CTD of RNAPII and recruits Iws1 and the REF1/Aly mRNA export adaptor to facilitate mRNA export [38]. Iws1, which recruits the HYPB/Setd2 histone methyltransferase to the RNAPII elongation complex forms a megacomplex that affects mRNA export as well as the histone modification state of active genes in yeast [39]. Also noteworthy is the association of transcribed genes with the nuclear pore complex [40]–[43]. Our data, therefore, indicate that the human THO/TREX complex is another important factor in the coupling of transcription elongation with mRNA export.

### Human THO/TREX is a key player for genome integrity

One key feature of the yeast THO complex is its functional relevance in maintaining genome integrity, in particular by limiting the co-transcriptional formation of R loops. A similar R-loop-dependent co-transcriptional genome instability is observed in mammalian cells with loss of the splicing factor ASF/SF2 [25], [44], [45]. A recent genome-wide siRNA screening performed to identify genes involved in genome stability by monitoring phosphorylation of the histone variant H2AX suggests that a specific class of RNA processing factors may help prevent genome instability [26]. In a number of cases the accumulation of γH2AX foci are suppressed by RNase H overexpression, as would be expected if they were mediated by R-loops, whether completely or partially. The relevance of R-loops in the origin of chromosome instability has been studied at the S regions of the Immunoglobulin genes of B cells. In this case the R-loop provides the substrate for the action of the AID deaminase, which specifically acts on the ssDNA displaced by the DNA∶RNA hybrid [33]. Interestingly, an involvement of THO in genome instability had not been shown in humans. This is of key importance, as it would clarify whether human THO/TREX functions *in vivo* during transcription to prevent R-loop formation and whether its function would be related to the co-trancriptional formation of an mRNP. Our study clearly shows an increase in DNA damage, as determined as a larger percentage of cells with γH2AX and 53BP1 foci, in cells depleted of human THO/TREX (Figure 2 and Figure S2).

**Figure 10:**
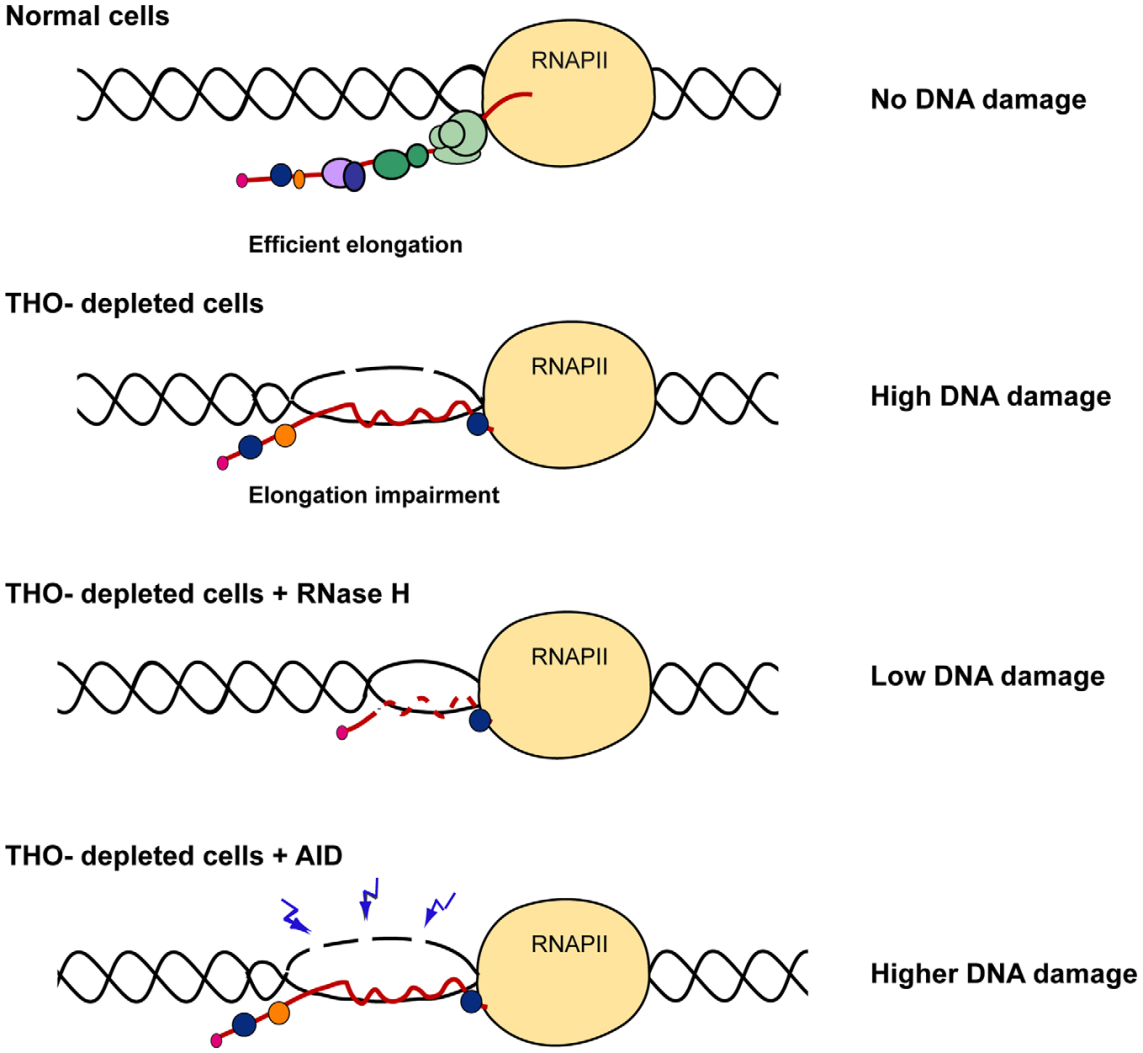
Model to explain the role of THO/TREX in the prevention of R-loop formation. THO/TREX contributes to the co-transcriptional formation of an optimal mRNP particle preventing hybridization of the nascent mRNA with the DNA template and formation of an R-loop. In THO-depleted human cells, R-loops are formed leading to a single-stranded DNA that is more susceptible to be damaged spontaneously by genotoxic agents or by AID. R-loop removal by RNase H over-expression would alleviate DNA damage and genome instability caused by THO depletion.

Accumulation of γH2AX foci of THO-depleted HeLa cells is suppressed by overexpresssion of RNAse H (Figure 7A) and enhanced by overexpression of the human cytidine deaminase AID (Figure 7B). These results are explained by the formation of DNA∶RNA hybrids, implying that the nascent mRNA could interact with the transcribed region behind the advancing RNAPII in the absence of human THO. This analysis demonstrates that indeed THO prevents R-loop formation in human cells (Figure 10). It is worth noting that a previously reported genome-wide analysis [26] failed to identify THO/TREX components among the affected RNAi-depleted cells leading to the accumulation of DNA breaks. THOC1 and THOC2-depleted cells appeared as showing a low proportion of cells (2–2.7%) with γH2AX foci. We believe that this could be due to the limitation of such a genome wide-analysis on cells depleted of essential factors that strongly affects their proliferation capacity, as is the case of THO-depleted human cells.

High levels of DNA breaks have been determined with the comet assay in cell lines depleted of different subunits of the THO/TREX complex (Figure 3A, 3B). The high accumulation of DNA breaks correlated with a hyper-recombination effect observed in the direct-repeat recombination construct pIREC after THOC1 depletion (Figure 6). Such a hyper-recombination phenotype was in any case lower than that of yeast THO mutants, which may be due to the fact that DSBs in mammals are more efficiently repaired via Non Homologous End Joining. Class switching, which is linked to transcription and R-loop formation at the S regions of the Ig genes [33], increased significantly in murine CH12 cells transfected with different siRNAs against THOC1 (Figure 8), consistent with a function of human THO preventing formation of R-loops and DSBs. Interestingly, THOC5 has recently been re-isolated in a screening of genes with a potential effect in CSR [46]. Altogether these data suggest that THO could play a role during normal B cell development, although further *in vivo* analysis would be needed to confirm this possibility. THO could contribute to the mRNP packaging of S regions. However, the structure and the G-richness of these S regions might make them difficult to assemble as an optimal ribonucleprotein even in the presence of THO. Consequently a basal level of R-loops could form at the S regions and promote the events necessary for normal development [9].

Finally, consistent with the idea that DNA breaks in THO-depleted human cells are linked to replication failures, we provide evidence in this study of an alteration in the pattern of replication in THOC1 depleted cells determined by DNA combing (Figure 9). This is consistent with the observation that transcription-associated recombination (TAR), as most forms of homologous recombination, is highly dependent on replication both in yeast and mammalian cells [47]–[49]. Highly transcribed genes are impediments for replication fork progression [50] and TAR may be linked to collisions between DNA replication and transcription machineries [51]. Thus, Topoisomerase I suppresses genome instability in mammalian cells by preventing conflicts between transcription and DNA replication [52]. Interestingly, however, our DNA combing analyses show that replicons seem longer. One possibility could be a putative incapacity of THOC1-depleted cells to trigger the S-phase or DNA damage response checkpoint and/or an incapacity to finish replication properly, leading to an apparent higher speed and longer replicons of THOC1-depleted cells. Indeed, yeast THO mutants activate the S-phase checkpoint and require an active S-phase checkpoint for viability under replicative stress [53]. Another plausible explanation of the longer replicons could be a reduction in the levels of transcribing RNAPII on the DNA, due to abortive transcription elongation. Faster replication forks have been detected for yeast *sgs1Δ* cells, which also show hyper-recombination [54]. Further molecular analyses of replication in THO-depleted human cells would be needed to understand the molecular basis for the DNA combing pattern observed.

In summary, our work shows that human THO controls transcription elongation at the interface with RNA processing and export, implying a physical connection with active chromatin. THO prevents the formation of R-loops that can compromise genome integrity by altering replication progression and leading to an accumulation of recombinogenic DNA breaks (Figure 9). This works, therefore, provides experimental evidence for a role of mRNP biogenesis factors in genome integrity in humans and reveals that the functional interconnection between mRNP biogenesis and the maintenance of genome integrity is more conserved than previously anticipated.

## Materials and Methods

### Antibodies

Commercial antibodies used were anti-ß actin, anti-THOC1 (Abcam), anti-γH2A (clone JBW301 Upstate), anti-53BP1 (NB100-304 Abyntec Biopharma), and mouse and rabbit polyclonal antibodies. For immunobloting, anti-mouse or anti-rabbit antibodies conjugated with horseradish peroxidase were used as secondary antibodies.

### Plasmids

pSUPER-RETRO GFP was used to clone specific DNA sequences for shRNA with *Bgl*II and *Hin*dIII and as indicated by the manufacturer (OligoEngine VEC-PRT-0005/0006). The TAN 1 system and the method for the measurement of luciferase and renilla activities have been described [28]. pcDNA6/TR (Invitrogen) and pTER [55] were used to generate stable inducible shRNA cells. pcDNA3-RNaseH1 and pcDNA3-RNaseH2 were kindly provided by F. Baas [56]. For the plasmid pIREC, the mutated EGFP from the vector pI [57] was replaced for GFP repeats generated by PCR. pcDNA3 (Invitrogen) was used to clone the open reading frame of human AID.

### Cell cultures and transfection

All cell lines used in this study, except CH12, were maintained in DMEM (Gibco) supplemented with 10% heat-inactivated fetal bovine serum at 37°C (5% CO_2_). Transient transfection of plasmid (4 µg) or siRNA (100 nM) was performed using Lipofectamine 2000 (Invitrogen, Carlsbad, CA) according to the manufacturer's instructions.

HeLa stable cell lines with THOC1 shRNA were established by Lipofectamine 2000-mediated transfection of pTER-THOC1, a TetR-expressing construct, pCDNA6TR, followed by selection with 5 µg/ml blasticidin and 100 µg/ml of zeocin. The two positive clones selected were named HeTH-1 and HeTH-4. The shRNA target sequence is available upon request. CH12 cell line was maintained in RPMI 1640 supplemented with 10% FBS, 10 mM of 2-mercaptoetanol and 5% NCTC (Invitrogen).

HeRG stable cell lines were established by Lipofectamine 2000-mediated transfection of the pIREC plasmid in a HeLa stable cell line carrying pcDNA6TR (a TetR-expressing construct), followed by selection with 5 µg/ml blasticidin and 500 µg/ml of G418. The construction was verified treating the cells with different drugs as camptotecyn and neocarzinostatin.

### Analysis of apoptosis

Hypodiploid apoptotic cells were detected by flow cytometry according to published procedures [58]. Basically, cells were washed with phosphate-buffered saline (PBS), fixed in cold 70% ethanol, and then stained with propidium iodide while treating with RNase. Quantitative analyses of sub-G_1_ cells were carried out in a FACScan cytometer using the Cell Quest software (BD Biosciences).

### Real-time qPCR

cDNA was synthesized from cytoplasmic RNA (1 µg) by reverse transcription using Super-Script TM First strand synthesis for RT-PCR (Invitrogen) and random primers. RT-qPCR was performed with SYBR qPCR Mix (Applied Biosystems) and analyzed on an ABI Prism 7000 (Applied Biosystems, Carlsbad, CA). Primers sets for this analysis are described in Table S1.

### Genome-wide gene expression analysis

Six independent microarray expression experiments were conducted. Affymetrix array experimental procedures were performed according to manufacturer's instructions at the CABIMER's Genomic Unit. Human Gene 1.0 ST array (Affymetrix, Santa Clara, USA) were used. The probe set signals were calculated using the Affymetrix GeneChip Operating Software 1.4.0.036. Linear fold-change cutoffs were analyzed at 95% confidence levels (p-values<0.05) in 1.5-fold down-regulated or up-regulated genes of THOC1-depleted cells. The microarray data were submitted to Gene expression Omnibus (GEO; accession number: GSE27091).

### Immunofluorescence *in situ* analysis

Cells cultured on glass coverslips were transfected with siRNA or plasmids (30% or 60–80% of confluency, respectively). After transfection, cells were cultured for 48 h, fixed in 2% formaldehyde in phosphate-buffered saline (PBS) and treated with Ethanol 70% for 5 min at −20°C, 5 min at 4°C, and washed twice in PBS. After blocking with 3% bovine serum albumin (BSA) in PBS, the coverslips were incubated with primary antibodies in 3% BSA in PBS followed by secondary antibodies conjugated withTexas Red goat anti-mouse or Alexa Fluor 568 goat anti-rabbit (Invitrogen). DNA was stained with DAPI.

### RNA fluorescence *in situ* hybridization

RNA *in situ* hybridization was carried out as described [20]. Cells cultured on glass coverslips were either transfected with plasmid shRNA or treated with doxycycline (in the case of stable shRNA clones). Samples were processed after 48 h and 96 h respectively. Cells were fixed in 4% formaldehyde in phosphate-buffered saline (PBS). Quantitation of the nuclear-cytoplasmic distribution of poly(A)+ RNA was done using the Multiwavelength-MetaMorph v7.5.1.0. software. The cellular periphery was defined with phase contrast images and the nucleus with DAPI staining. The cytoplasmic: nuclear ratio of the mean fluorescence intensities was determined. All experimental analyses were performed with 7.5 10^4^ cells for both HeLa or HeTH-4 cell lines

### Comet assay

DNA DSBs were analyzed using a commercial comet assay (Trevigen, Inc.) following the manufacturer's protocol. For quantification, comet-positive cells were scored in random fields of cells. More than 100 cells from each sample were scored. The quantitative analysis was performed with the Comet-score software (version 1.5).

### Class-switching measurements

CH12 cells were transfected with pSUPERshRNA targeting THOC1 using Nucleofector (Amaxa). CH12 cells were stimulated for 12 h by adding 1 ng/ml of TGFB (R&D Systems), 5 ng/ml of IL4 (Bionova) and 0.5 µg/ml of anti-CD40 (Pharmigen). Surface IgA was stained with anti-mouse IgA-RPE antibody (AbDSerotec) and analyzed by flow cytometry 72 h after transfection. Double positive GFP-RPE cells were counted.

### DNA combing

DNA combing was performed as described [59]. Briefly, DNA fibres were extracted from cells in agarose plugs immediately after CldU labeling and were stretched on silanized coverslips. DNA molecules were counterstained with an anti-ssDNA antibody (MAB3034, Chemicon; 1/500) and an anti-mouse IgG coupled to Alexa 546 (A11030, Molecular Probes, 1/50). CldU and IdU were detected with BU1/75 (AbCys, 1/20) and BD44 (Becton Dickinson, 1/20) anti-BrdU antibodies, respectively. DNA fibres were analysed on a Leica DM6000 microscope equipped with a DFC390 camera (Leica). Data acquisition was performed with LAS AF (Leica). Representative images of DNA fibers were assembled from different microscopic fields of view and were processed as described [60].

## Supporting Information

Figure S1 Analysis of transcription defects in THO/TREX depleted cells. A) THO/TREX depletion impairs gene expression. HPRT expression determined by RT-PCR after 96 h of depletion with siRNAs (hHpr1/THOC1, THOC5, UAP56 and ALY). α-amanitin was used as a positive control of transcription inhibition. B) qRT-PCR analysis of the mRNA levels of FLUC is shown in the upper panel and the FLUC∶RLUC ratio of mRNA levels in cells depleted of different THO/TREX subunits is shown below. Other details as in Figure 1.
(TIF)

Figure S2 shRNA interference of THO/TREX stimulates the cellular DNA damage response. A) Relative expression of THO/TREX components after shRNA transfections is shown. HeLa cells were transiently transfected with a pSUPER vector for shRNA expression that carries a GFP gene reporter. shTM was used as a control (for more details see Materials and Methods). B) Quantification of γ-H2AX and 53BP1 foci in GFP positive cells. C) Immunofluorescence of γH2AX and 53BP1 48 h after transfection with the indicated shRNAs. Nuclei were stained with DAPI. Other details as in Figure 2.
(TIF)

Figure S3 Effect of THOC1 and THOC5 depletion in DNA damage response in MRC5 cells. Quantification of the tail moment at 72 h after siRNA depletion. Error bars indicate standard errors of the mean from three independent experiments. Other details as in Figure 3.
(TIF)

Figure S4 Depletion of THOC1 affects growth rate in HeTH cells. Growth rate of HeTH-1 and HeTH-4 in the presence or absence of doxycycline.
(TIF)

Figure S5 Effect of α-amanitin in transcription of PTBP1. Effect of α-amanitin treatment in transcription of the endogenous gene PTBP1 as determined by RT-qPCR. The relative amount of nascent mRNA in HeTH-4 cells is plotted. The cells were treated with 5 µg/ml of α-amanitin (24 hours before collecting cells for RNA extraction); other details as in Figure 4.
(TIF)

Figure S6 Quantitative PCR analysis for Iμ and AID transcripts in unstimulated and stimulated CH12 cells. mRNA levels were normalized respect to CD3 expression levels.
(TIF)

Figure S7 DNA combing assay in the stable cell line HeTH4. Replication fork velocity in the presence or absence of doxycycline. Other details as in Figure 9.
(TIF)

Table S1 Table of Primers: The name and the sequence of primers used in Real-Time qPCR analyses are shown.
(TIF)

Table S2 Deregulated genes in THOC1 depleted cells (HeTH-4+DOX). THOC1-depleted cell genome-wide gene expression profile was analyzed on a high density oligonucleotide microarray (Human Gene 1.0 ST arrays, Affimetrix, Santa Clara, SA). Down-regulated and up-regulated well-annotated genes with ≥1.5 linear fold change and p-values≤0.05 are shown.
(TIF)
