# Supplementary figures and images for: Genome Instability and Transcription Elongation Impairment in Human Cells Depleted of THO/TREX
Source: PLoS Genet. 2011 Dec 1;7(12):e1002386. doi: 10.1371/journal.pgen.1002386 (PMC3228816; doi:10.1371/journal.pgen.1002386)

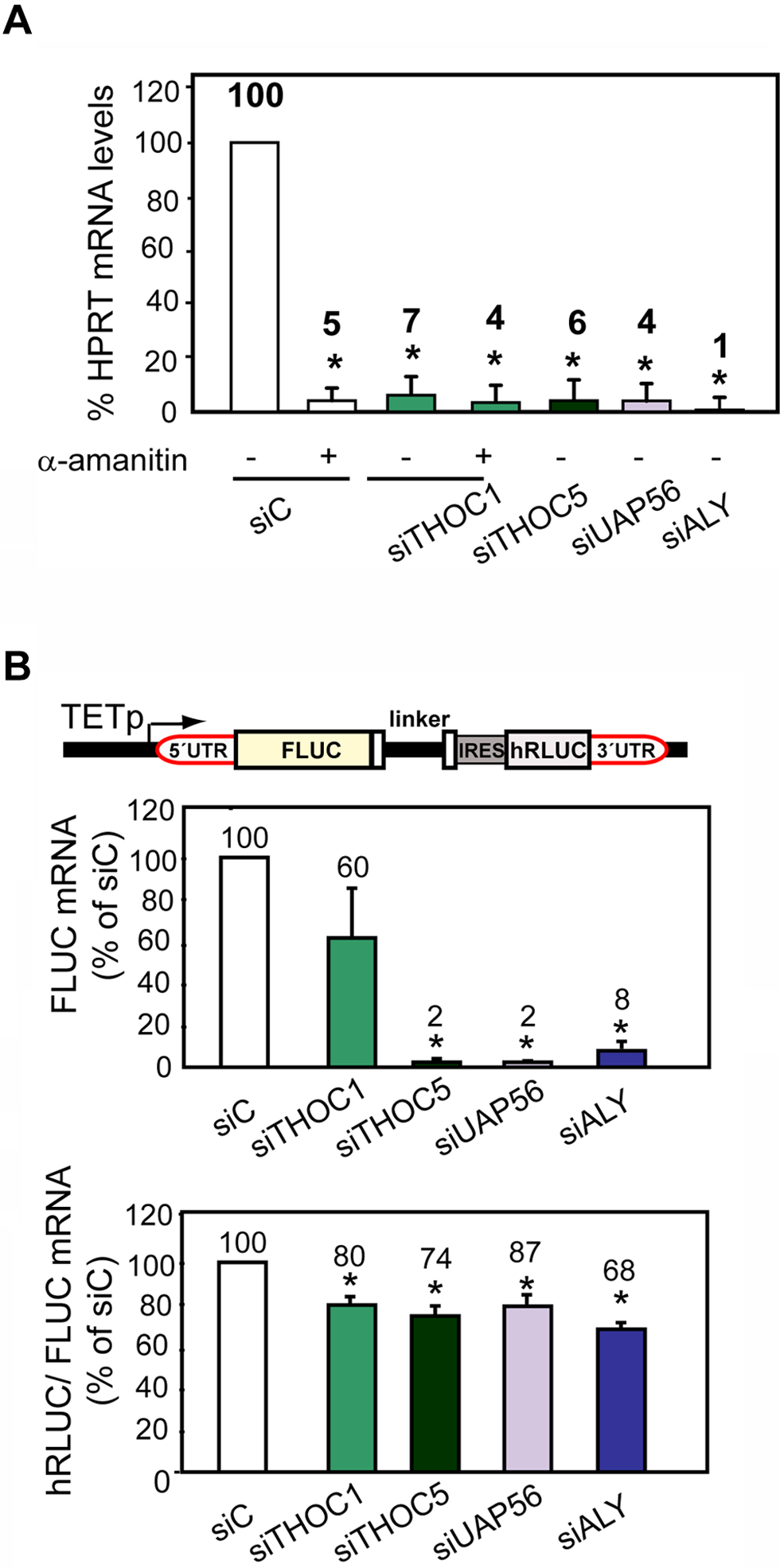

Supplement: Figure S1 — Analysis of transcription defects in THO/TREX depleted cells. A) THO/TREX depletion impairs gene expression. HPRT expression determined by RT-PCR after 96 h of depletion with siRNAs (hHpr1/THOC1, THOC5, UAP56 and ALY). α-amanitin was used as a positive control of transcription inhibition. B) qRT-PCR analysis of the mRNA levels of FLUC is shown in the upper panel and the FLUC∶RLUC ratio of mRNA levels in cells depleted of different THO/TREX subunits is shown below. Other details as in Figure 1. (TIF) [file pgen.1002386.s001.tif]

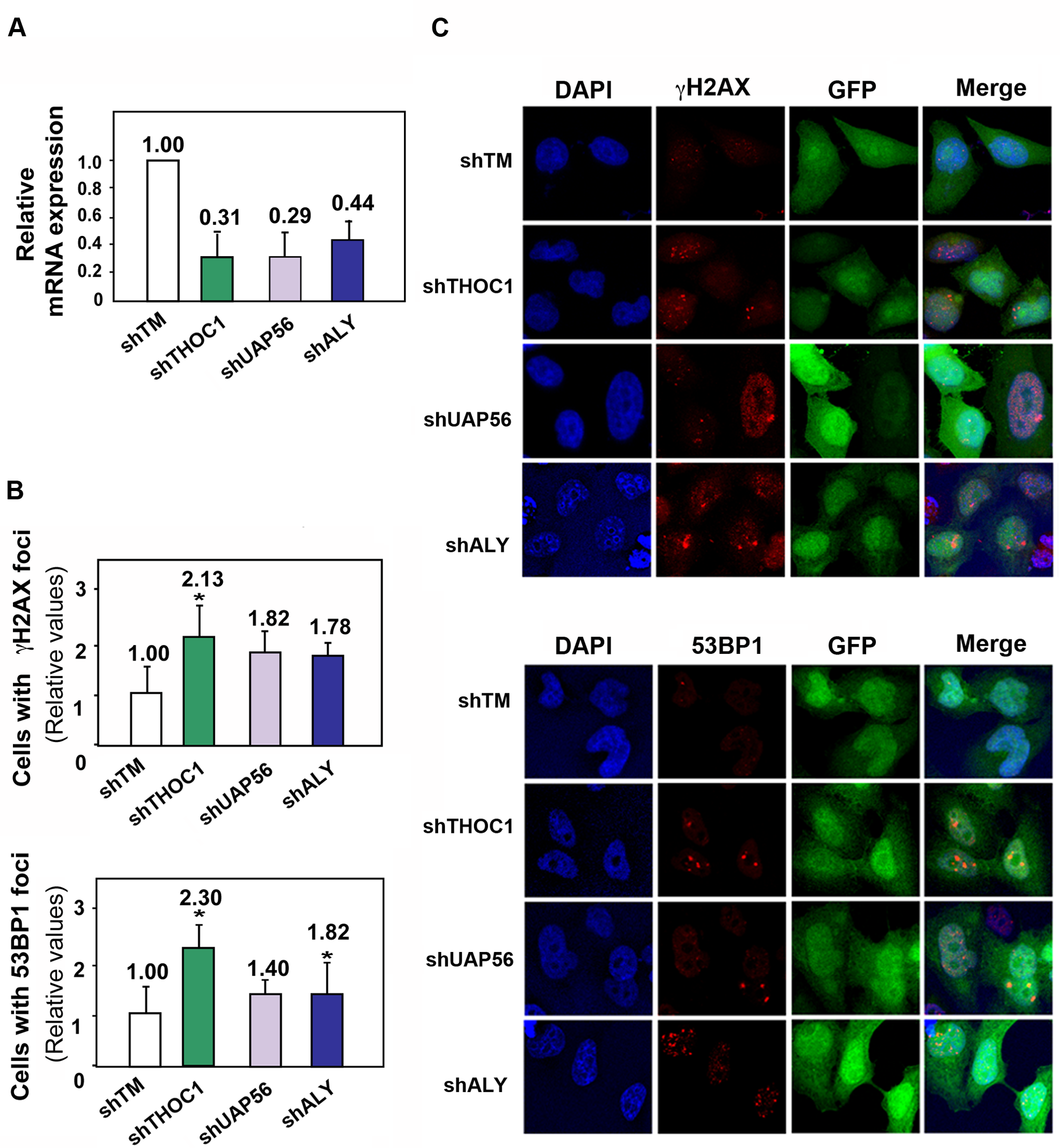

Supplement: Figure S2 — shRNA interference of THO/TREX stimulates the cellular DNA damage response. A) Relative expression of THO/TREX components after shRNA transfections is shown. HeLa cells were transiently transfected with a pSUPER vector for shRNA expression that carries a GFP gene reporter. shTM was used as a control (for more details see Materials and Methods). B) Quantification of γ-H2AX and 53BP1 foci in GFP positive cells. C) Immunofluorescence of γH2AX and 53BP1 48 h after transfection with the indicated shRNAs. Nuclei were stained with DAPI. Other details as in Figure 2. (TIF) [file pgen.1002386.s002.tif]

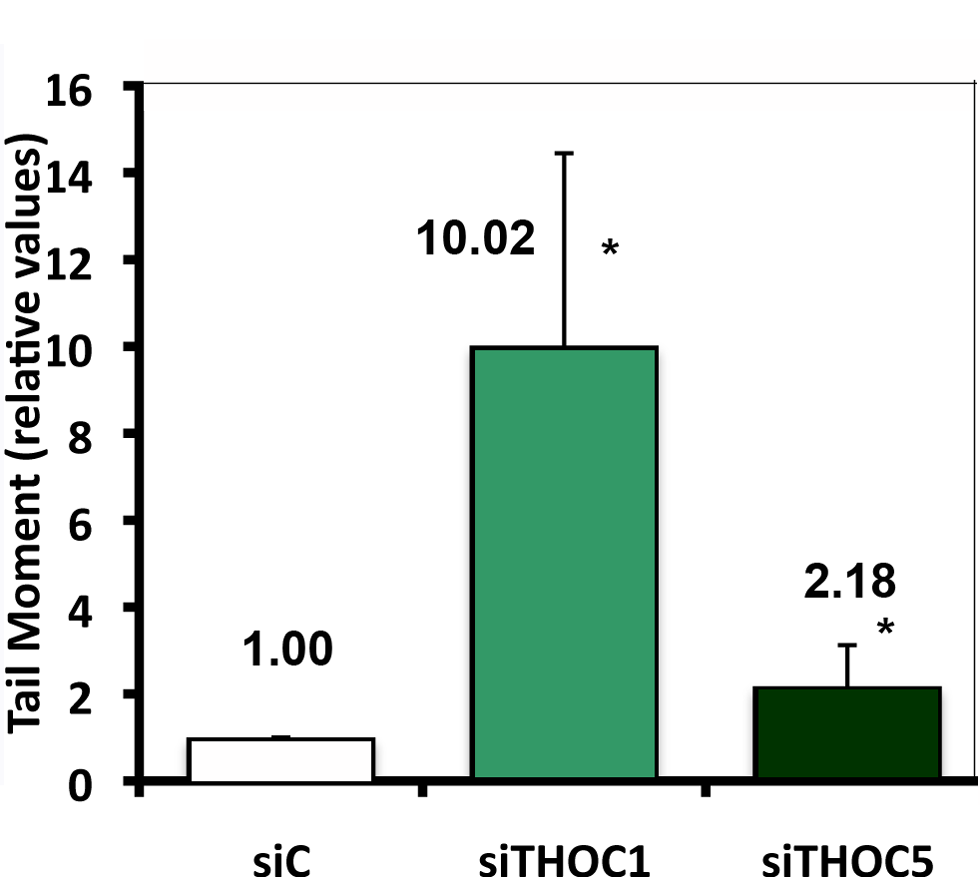

Supplement: Figure S3 — Effect of THOC1 and THOC5 depletion in DNA damage response in MRC5 cells. Quantification of the tail moment at 72 h after siRNA depletion. Error bars indicate standard errors of the mean from three independent experiments. Other details as in Figure 3. (TIF) [file pgen.1002386.s003.tif]

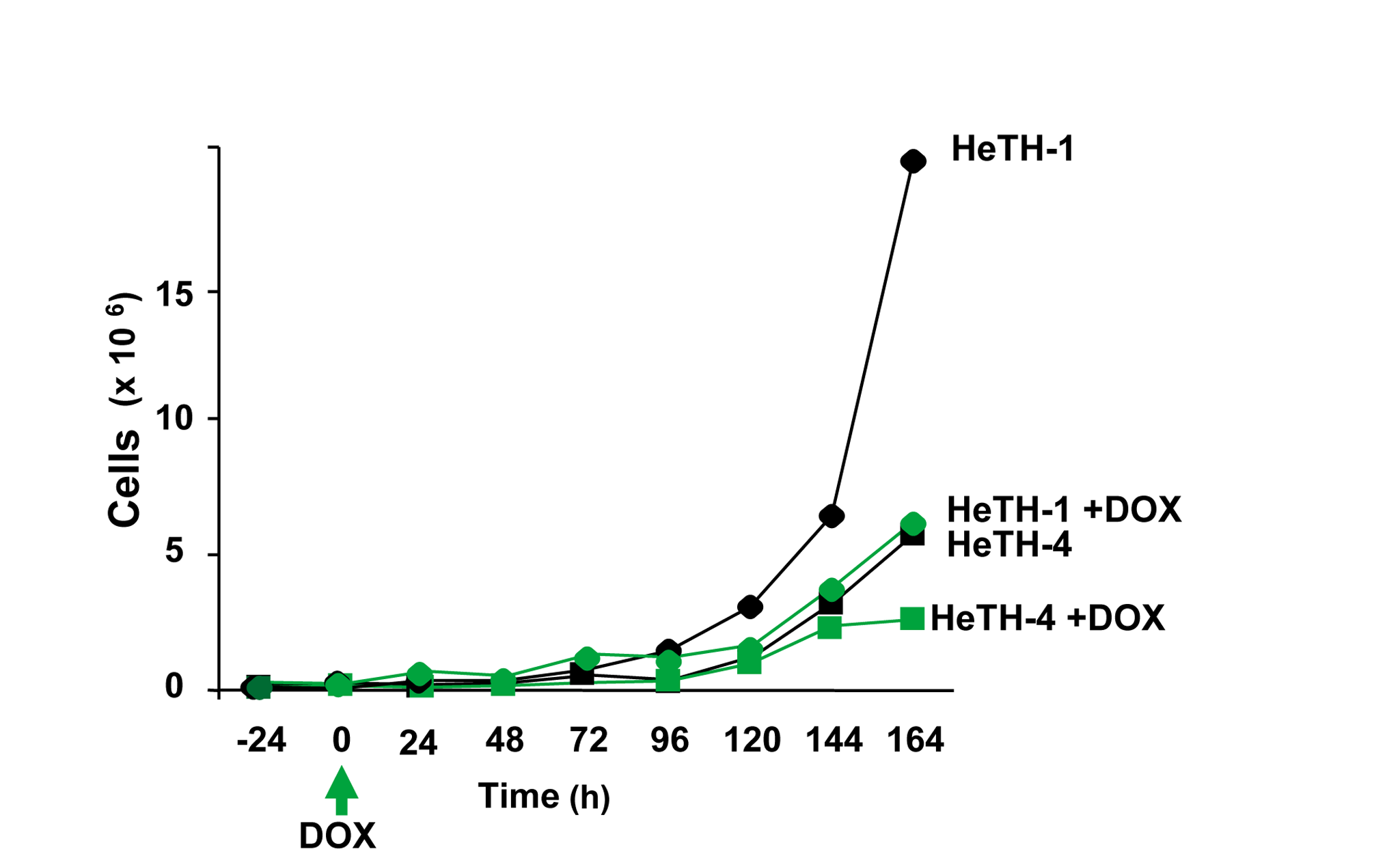

Supplement: Figure S4 — Depletion of THOC1 affects growth rate in HeTH cells. Growth rate of HeTH-1 and HeTH-4 in the presence or absence of doxycycline. (TIF) [file pgen.1002386.s004.tif]

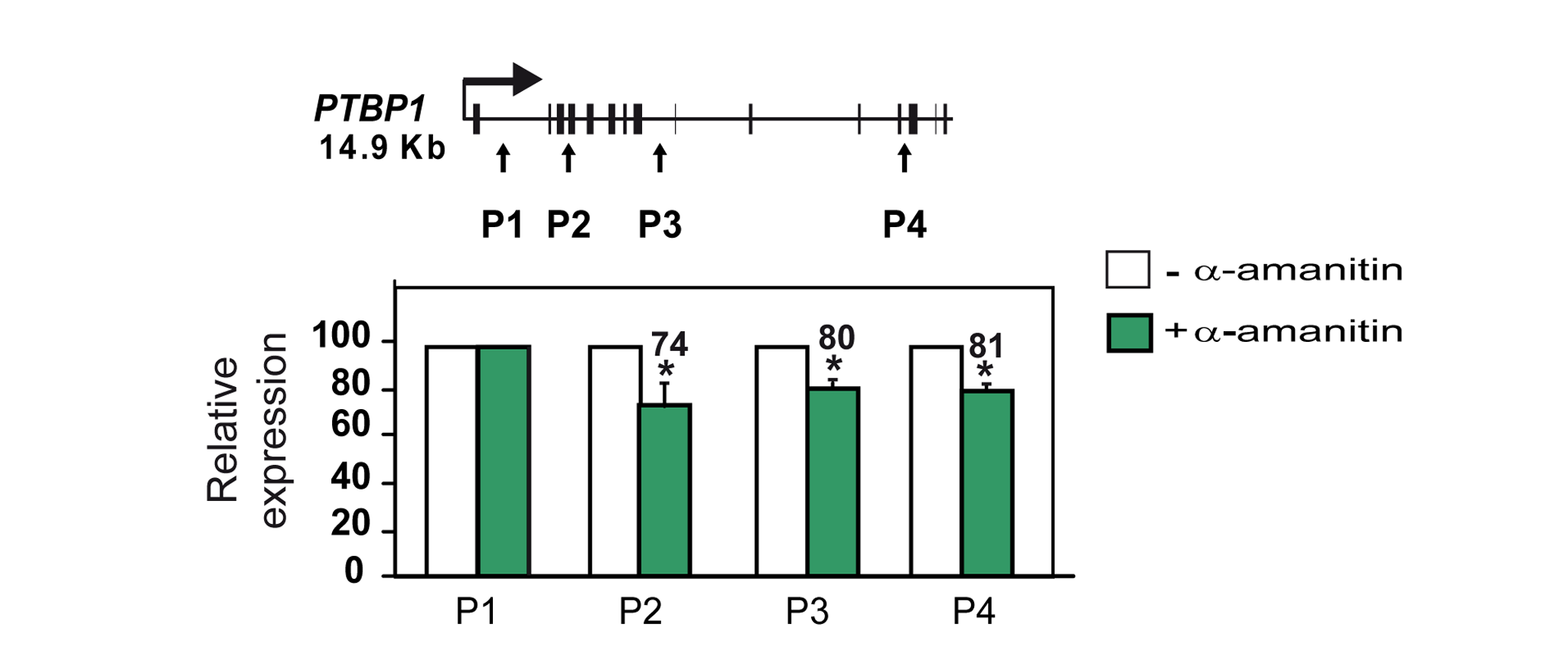

Supplement: Figure S5 — Effect of α-amanitin in transcription of PTBP1. Effect of α-amanitin treatment in transcription of the endogenous gene PTBP1 as determined by RT-qPCR. The relative amount of nascent mRNA in HeTH-4 cells is plotted. The cells were treated with 5 µg/ml of α-amanitin (24 hours before collecting cells for RNA extraction); other details as in Figure 4. (TIF) [file pgen.1002386.s005.tif]

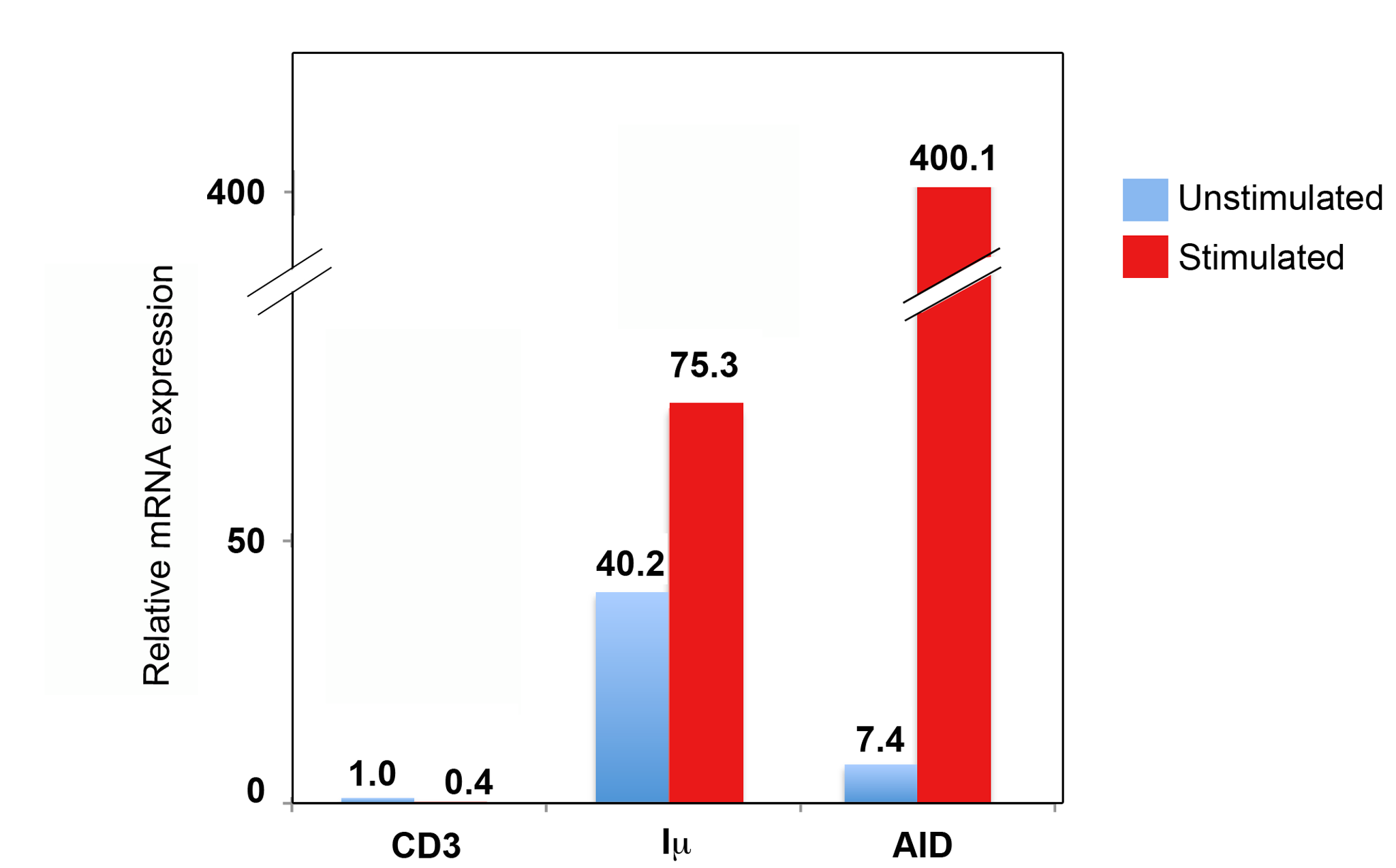

Supplement: Figure S6 — Quantitative PCR analysis for Iμ and AID transcripts in unstimulated and stimulated CH12 cells. mRNA levels were normalized respect to CD3 expression levels. (TIF) [file pgen.1002386.s006.tif]

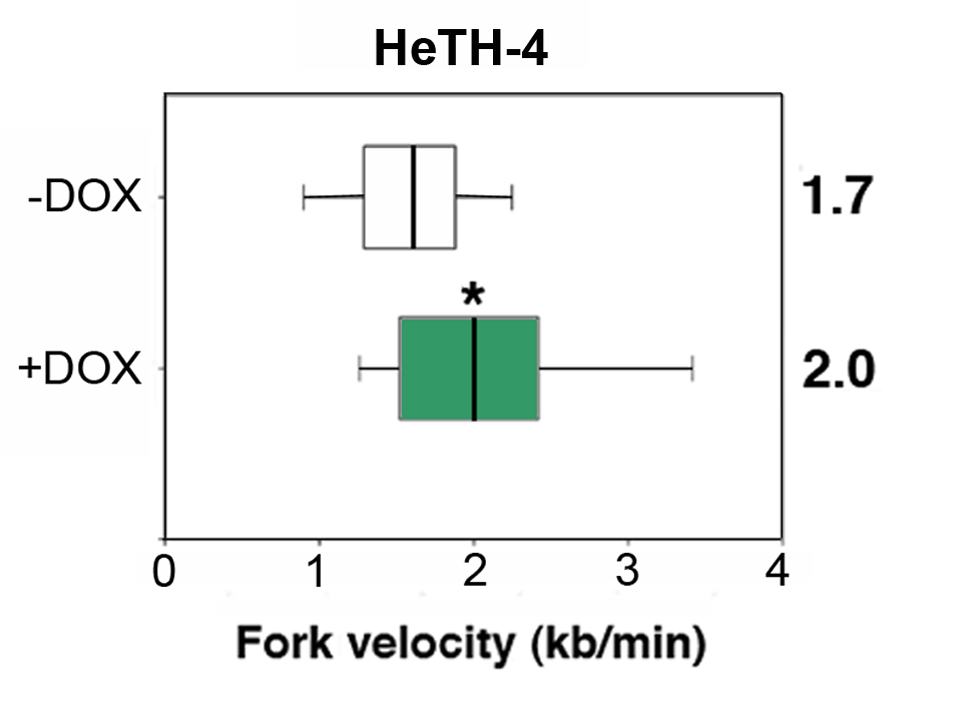

Supplement: Figure S7 — DNA combing assay in the stable cell line HeTH4. Replication fork velocity in the presence or absence of doxycycline. Other details as in Figure 9. (TIF) [file pgen.1002386.s007.tif]

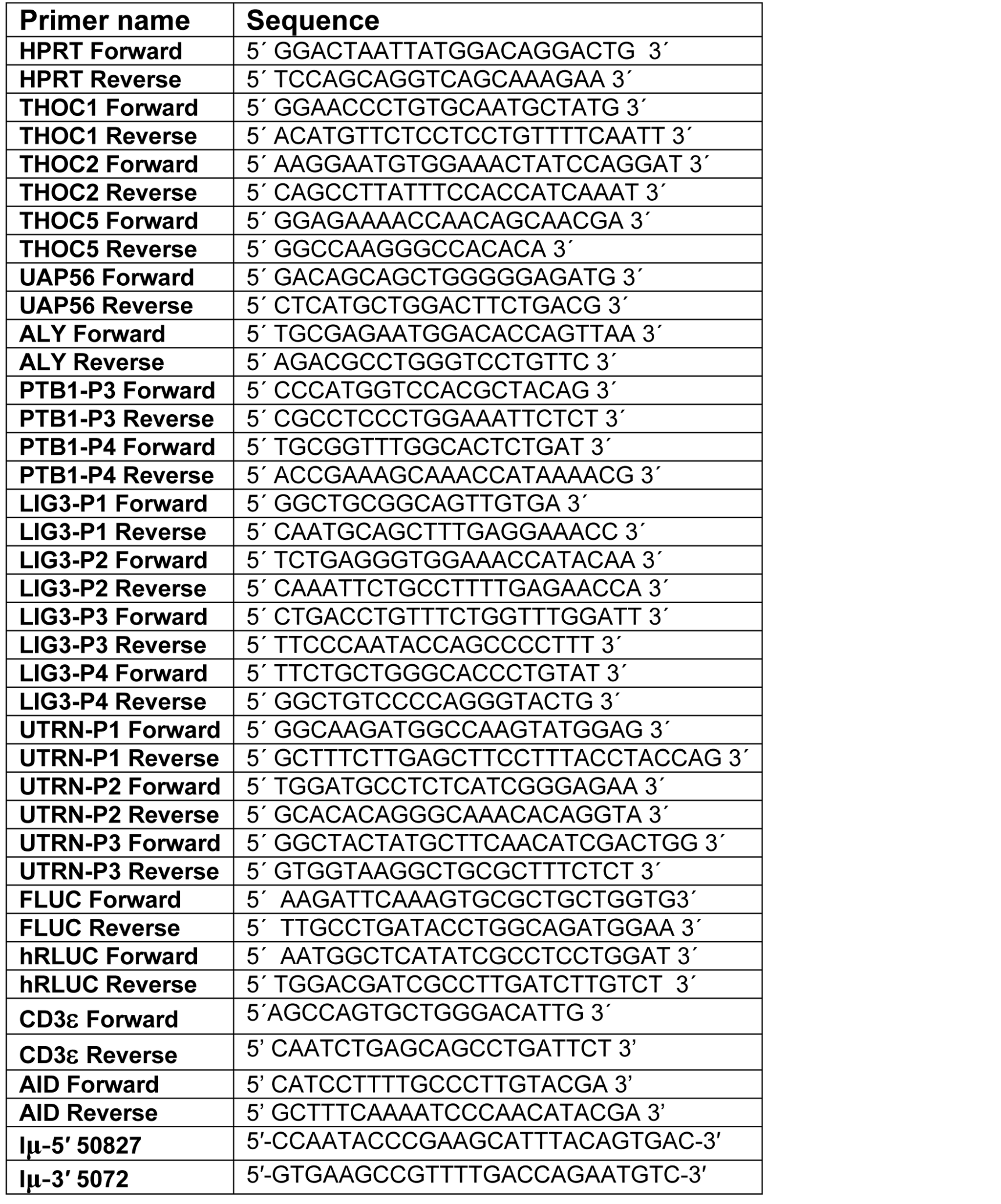

Supplement: Table S1 — Table of Primers: The name and the sequence of primers used in Real-Time qPCR analyses are shown. (TIF) [file pgen.1002386.s008.tif]

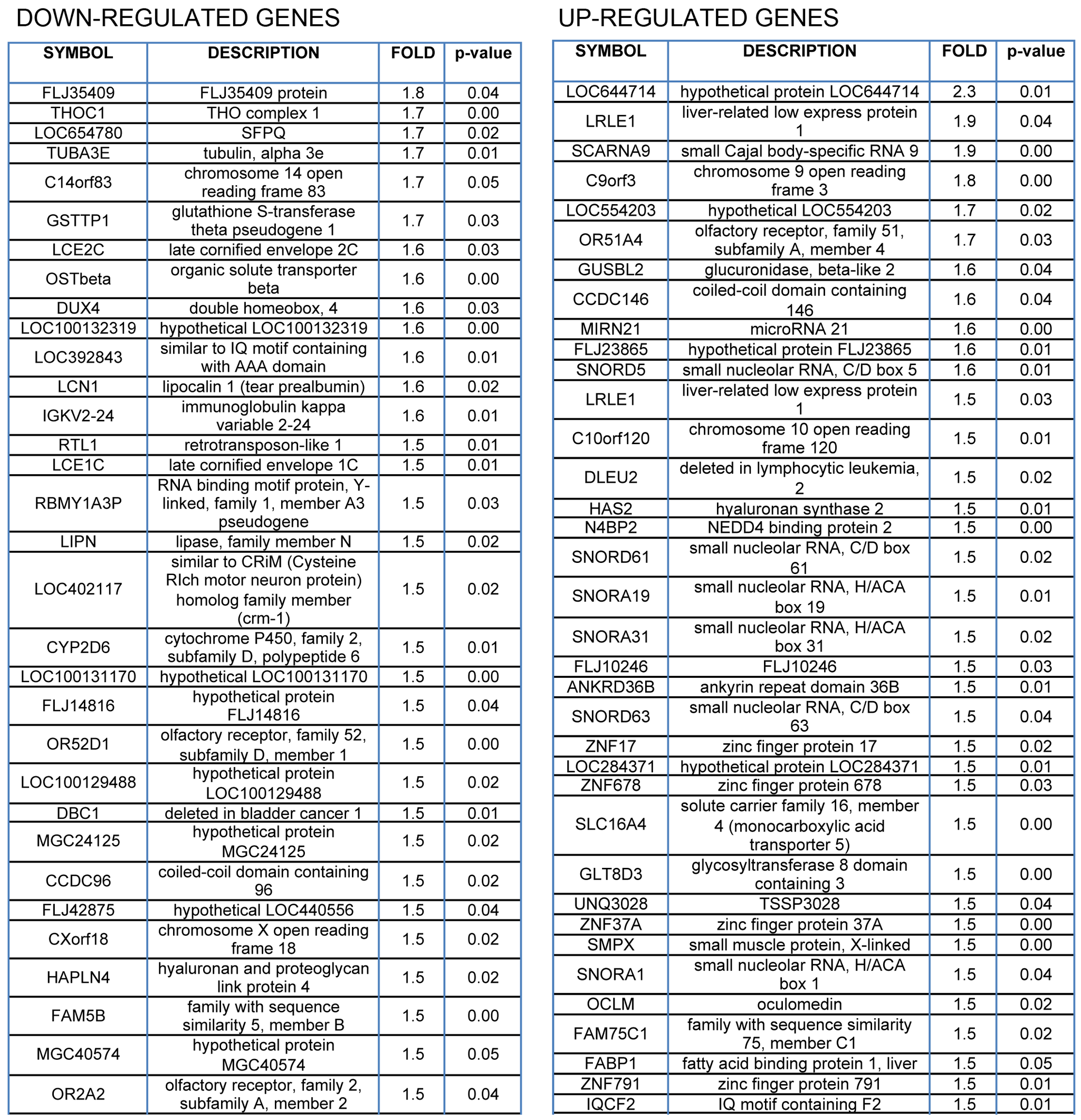

Supplement: Table S2 — Deregulated genes in THOC1 depleted cells (HeTH-4+DOX). THOC1-depleted cell genome-wide gene expression profile was analyzed on a high density oligonucleotide microarray (Human Gene 1.0 ST arrays, Affimetrix, Santa Clara, SA). Down-regulated and up-regulated well-annotated genes with ≥1.5 linear fold change and p-values≤0.05 are shown. (TIF) [file pgen.1002386.s009.tif]
